# Theoretical and Experimental Investigation of Shape Memory Polymers Programmed below Glass Transition Temperature

**DOI:** 10.3390/polym14132753

**Published:** 2022-07-05

**Authors:** Kartikey Shahi, Velmurugan Ramachandran

**Affiliations:** Department of Aerospace Engineering, Indian Institute of Technology Madras, Chennai 600036, India; kartikeyshahi.iitm@gmail.com

**Keywords:** shape memory polymers, smart materials, large deformation, constitutive modeling, cold programming

## Abstract

An epoxy-based shape memory polymer (SMP) is synthesized and examined for its deterioration in shape fixity due to springback and isothermal viscoelastic recovery at different ambient temperatures. Shape fixity depends not only on material properties but also on programming conditions. A constitutive finite deformation model is incorporated to predict the behavior of the proposed SMP and find maximum shape fixity. A programming approach is followed in which, in contrast to hot programming, the SMPs are neither heated before deformation nor cooled afterward but are deformed at ambient temperature and then stress-relaxed. The proximity of the programming temperature to the glass transition temperature plays a crucial role in determining the shape fixity of SMP. It has been found that the SMP with a glass transition temperature of 42.9 °C can achieve maximum shape fixity of 92.25% when programmed at 23 °C with 100 min stress relaxation time. Thermal contraction and dynamic tests are performed in the Dynamic Mechanical Analyzer (DMA) to determine structural relaxation properties and distinguish the programming temperature in the cold, warm or hot temperature zone. The shape memory tests are carried out using temperature-controlled UTM to determine the shape fixity and shape recovery of SMP. The SMPs are subjected to a full thermomechanical cycle with different stress relaxation times and programming temperatures.

## 1. Introduction

Shape Memory Polymers (SMPs) are a class of smart materials that exhibit shape retention and recovery behavior. Most heat-shrinkable polymers show some shape memory effect, but their use depends entirely on the application. In addition to heat, different SMPs can respond to other stimuli such as magnetic field, electricity, light, chemicals, and moisture [1,2,3,4,5,6,7]. The ability of SMPs to recover large deformations, heal microdamages and act as actuators with the least mechanical complexity has also increased its scope in the automotive, aerospace, and aeronautical sectors [8,9,10,11,12,13,14,15]. Recent developments include SMP solar cells that can be folded compactly for installation on spacecraft and deployed in space [16]. In the medical field, it can be used as stents, drug delivery systems, and stitches [17]. Furthermore, the SMPs are used as nanostructures to tune the adhesive superhydrophobicity of solid/liquid contact states [18]. In addition, two-stage reactive SMPs that possess better mechanical and thermal properties have potential applications for robust 3D part design [19]. Both thermoplastic and thermoset polymers have been used for shape memory applications; however, thermoset has the advantage of high strength and temperature applications [20]. Current work is limited to epoxy-based thermoset SMPs that undergo large plastic deformations during the programming stage.

The conventional programming technique for imparting a new shape is to heat the amorphous SMP above its glass transition temperature $T_{g}$, deform it, hold the deformation, and cool it. This method is called hot programming [21]. The phase transition while cooling is assumed to impart a shape memory effect by storing elastic energy in a frozen state that can be recovered later by reheating. Recent advances in programming have emphasized avoiding the heating and cooling steps rather than achieving a new shape at room temperature by cold drawing or compression followed by stress relaxation [21,22,23,24,25]. The main requirement for an amorphous SMP to retain deformation at room temperature is that the deformation must be large enough to cause yielding and plastic flow; otherwise, the SMP would revert to its original shape [26]. In other words, for cold programming, the SMP must be synthesized such that it exhibits viscoelastic-plastic behavior at the desired strain. The proximity of room temperature to the glass transition temperature plays a crucial role in deciding whether the SMP is brittle or ductile in nature. Xie et al. (2009) [27] proposed an SMP made of Neopentyl Glycol Diglycidyl Ether (NGDE) and Diglycidyl Ether of Bisphenol A (DGEBA), which can be tailored for different $T_{g}$ by changing the proportion of constituents. NGDE as a flexible linear polymer not only makes epoxy (DGEBA) suitable for cold programming, it is also tolerant of damage.

Li et al. (2011) [22] performed cold compression of polystyrene-based thermoset SMP and studied the effect of ambient temperature (or programming temperature, $T_{p}$), strain and stress-relaxation time on shape fixity and shape recovery. Shape fixity was found to decrease with low programming strain and reduced stress relaxation time and increase with increasing programming temperature. Zotzmann et al. (2010) [28] ruled out the possibility of achieving a good shape memory effect with the cold drawing process, stating that thermoset SMPs cannot undergo large deformation without failing. To overcome this shortcoming of cold drawing, Abishera et al. (2016) [26] used a flexible polymer proposed by Xie et al. (2009) [27] and performed tensile rather than compression shape memory tests at room temperature. They were able to obtain good shape memory; however, they did not perform thermomechanical tests at different ambient temperatures. Both Li et al. (2011) [22] and Abishera et al. (2016) [26] used a four-step thermomechanical cycle, that is, deformation, stress-relaxation, instantaneous unloading, and heat recovery. Shahi et al. (2021) [25] investigated the loss of shape after the unloading step and found that if the ambient temperature of SMP subjected to cold drawing is close to $T_{g}$, the fixation of the shape will be less as the SMP tends to lose its shape over time. The present work is an extension of the aforementioned studies by including an additional step, which makes it a five-step thermomechanical cycle. The additional step is to observe the cold-drawn SMP for possible isothermal recovery after unloading at different ambient temperatures ($T_{p}$) in stress-free conditions. Li et al. (2016) [21] have also mentioned that isothermal viscoelastic recovery after the unloading can lead to poor shape fixation of cold programmed SMPs. The question arises of what the gap between $T_{g}$ and $T_{p}$ should be for maximum shape fixation. In this work, a methodology has been proposed to find the optimal temperature gap between $T_{g}$ and $T_{p}$. This required multiple experiments to be performed at close $T_{p}$ ranges, so a material simulation was performed to reduce the cost of experimentation.

Various approaches have been developed in the past to model the shape memory effect of amorphous polymers. These can be broadly classified as rheological [29,30,31] and phenomenological models [32,33,34,35]. The basic models are rheological models such as those proposed by Bhattacharyya et al. (2000) [30] that use friction elements to model irrecoverable low-temperature deformations. Liu et al.’s (2006) [33] constitutive phenomenological model considered the concept of “two phases”, that is, “frozen” and “active” phases. In the frozen phase, that is, the glassy phase, conformational rotation is totally locked, while in the active phase, that is, the rubber phase, the conformational movement can occur. The phase transition between frozen and active could capture fixity and shape recovery to a great extent. Nguyen et al. (2008) [36] proposed a mechanism-based model that incorporates structural relaxation and stress relaxation as the physics behind shape memory. This th changed the existing perception of the memory effect that the phase transition is mandatory for the fixation of the shape. This gave a reasonable explanation of how the shape can be set even at room temperature using cold programming without heating and then cooling the SMP. In simple words, if an SMP is plastically deformed and the stress is allowed to relax, the new shape is preserved due to the low mobility of the chain after unloading. Due to the greater mobility of the chain when heated, the elastic energy stored during loading is released so that SMP recovers its shape. Since this model could predict large deformations and the shape memory effect at room temperature, it was incorporated in the present study.

The aim of this work is to study the effect of programming temperature on shape fixity and to understand how it deteriorates when the programming temperature is chosen in the glass transition zone rather than in the glassy state. To achieve this goal, a SMP was synthesized using the material proposed by Xie et al. (2009) [27], and its thermal properties were tested using a Dynamic Mechanical Analyzer. Thermomechanical cycles of large deformations were carried out in temperature-controlled UTM with different programming conditions. The constitutive three-dimensional finite strain model proposed by Nguyen et al. (2008) [36] was simplified for one-dimensional uniaxial tensile loading, stress relaxation, unloading, isothermal recovery, and heated recovery. The material constants in the model were determined by curve simulating the experiments, and thermomechanical predictions were validated using experimental results. Finally, simulation was used to find the effect of various programming parameters on the shape memory properties of SMP.

## 2. Experimental Method

### 2.1. Polymer Selection and Specimen Preparation

The material proposed by Xie et al. (2009) [27] was chosen and synthesized with a unique constituent ratio. The constituents are a rigid aromatic diepoxide (Araldite LY556), i.e., Diglycidyl Ether of Bisphenol A (DGEBA), a flexible aliphatic diepoxide, i.e., Neopentyl Glycol Diglycidyl Ether (NGDE), and an aliphatic diamine crosslinker, i.e., Jeffamine 230. DGEBA:NGDE was approximately maintained about 13:7. Since Jeffamine’s active amine sites are responsible for the crosslinking between the DGEBA–DGEBA, DGEBA–NGDE, and NGDE–NGDE molecules, Equation (1) can be used to find its required weight. Approximately 100 g of final resin mix contained 46.85 g of DGEBA, 25.23 g of NGDE, and 27.92 g of Jeffamine. The mixture was thoroughly stirred on a magnetic stirrer and poured into silicone rubber molds. The mixture was then allowed to cure at room temperature for 24 h to obtain satisfactory mechanical properties, followed by post-cure for 3 h at 80 °C to complete the remaining polymerization. The equivalent weight values obtained from the supplier’s technical data sheet are $HEW_{Jeffamine\;230}$ = 57.5 g/eq, $EEW_{DGEBA}$ = 186 g/eq, and $EEW_{NGDE}$ = 108 g/eq.

(1) $$W_{Jeffamine\;230}=HEW_{Jeffamine\;230}\;\times \;\left(\frac{W_{DGEBA}}{EEW_{DGEBA}}+\frac{W_{NGDE}}{EEW_{NGDE}}\right)$$

where *EEW*—Epoxide Equivalent Weight (g/eq), *HEW*—Hydrogen Equivalent Weight (g/eq), and *W*—Weight (g).

### 2.2. Dynamic Mechanical Analysis

The phase transition was studied with the Dynamic Mechanical Analyzer, NETZSCH DMA 242 E. A tension-type specimen holder was used to hold the specimens with dimensions of 7.6 × 1.3 × 5.5 mm${}^{3}$. The DMA was operated in dynamic mode with a mixed stress–strain mode to apply a sinusoidal strain with an amplitude of 40 μm, keeping the maximum dynamic force at 3 N, the frequency at 1 Hz and the proportionality factor at 1.1. The first measurement segment was established as an isothermal at 10 °C for 30 min. Then, the temperature was raised from 10 to 80 °C with a rate of 4 °C/min.

### 2.3. Stress-Free Cooling

To obtain the stress-free cooling behavior of SMP, the creep mode in DMA was used with a negligible creep load of 0.001 N. Sample size and clamp type were the same as for the dynamic mode. The SMP was brought to an initial temperature of 0 °C, heated to 90 °C, held at 90 °C for 30 min and finally cooled again to 0 °C with a rate of 4 °C/min. The structural relaxation properties of SMP were measured by the cooling part of the measurements.

### 2.4. Uniaxial Tensile Tests

The uniaxial tensile tests were carried out in KALPAK temperature-controlled UTM in order to find the mechanical behavior of SMP in glassy, rubbery and glass transition phase. Temperature-dependent elastic and viscoplastic behaviors, such as modulus, distinct yield point, and strain softening, were measured using UTM. The samples were kept inside the chamber at the desired temperature for 30 min before performing the tensile tests. Temperature values were measured by a thermocouple placed near the sample and confirmed by taking readings through a non-contact digital infrared thermometer pointed at the surface of the sample. The crosshead speed was maintained at 2 mm/min for samples with dimensions according to ASTM 638D IV. The tests were carried out at temperatures ranging from 20 to 80 °C.

### 2.5. Shape Memory Tests

Shape fixity and recovery were tested by performing a five-step thermomechanical cycle in UTM with KALPAK temperature control. Strain was measured using a non-contact technique known as digital image correlation (DIC). The ASTM 638D IV samples were coated with white paint, and black paint was sprayed to create fine dots. The sample surface was visible to the chamber through the clear glass door of the temperature chamber. DIC was carried out on images captured through VIC 2D software. The first step was to load the samples to a particular deformation so that the desired level of strain was achieved. The deformation was paused while the samples were held in the grippers, and the stress relaxation was measured for 45 min in the second step. These two steps were carried out at the desired temperature, keeping the chamber doors closed. In the third step, the chamber doors were opened, and the lower gripper jaws were opened. The instantaneous springback recovery of strain was measured for 30 s, and the doors were closed immediately thereafter. It was assumed that the temperature of the sample did not change during the 30 s duration that the chamber doors were opened. In the fourth step, the sample was kept under observation for 45 min in a stress-free state and held only through the upper jaws. This step was performed to understand the effect of isothermal recovery on the shape fixity of the SMP. The final step was to heat SMP to regain shape. This was achieved by setting the final temperature at 80 °C. The heating rate was set at 4 °C/min. Several case studies were conducted by changing programming parameters such as stress relaxation time and programming temperature.

## 3. Constitutive Framework for Finite Deformation

A thermoviscoelastic model proposed by Nguyen et al. (2008) [36] is included in the present work. This model is capable of predicting shape fixity and shape recovery of SMPs programmed below the glass transition temperature. The new shape is fixed by relaxing the stress of deformed SMP without any heating and cooling steps. Therefore, the structural relaxation and stress relaxation processes are considered to model shape recovery and shape fixity, respectively. Structural rearrangements, also known as relaxation, occur in the polymer as a result of strain and temperature changes. Structural relaxation occurs when the polymer is subjected to an isothermal hold [37]. The material properties become time-dependent and gradually approach equilibrium. While in stress relaxation, when the polymer is subjected to a change in strain, the stress developed shows a time-dependent response [38]. Constitutive laws and solution methods are briefly discussed in the subsections.

### 3.1. Kinematics

**Figure 1:**
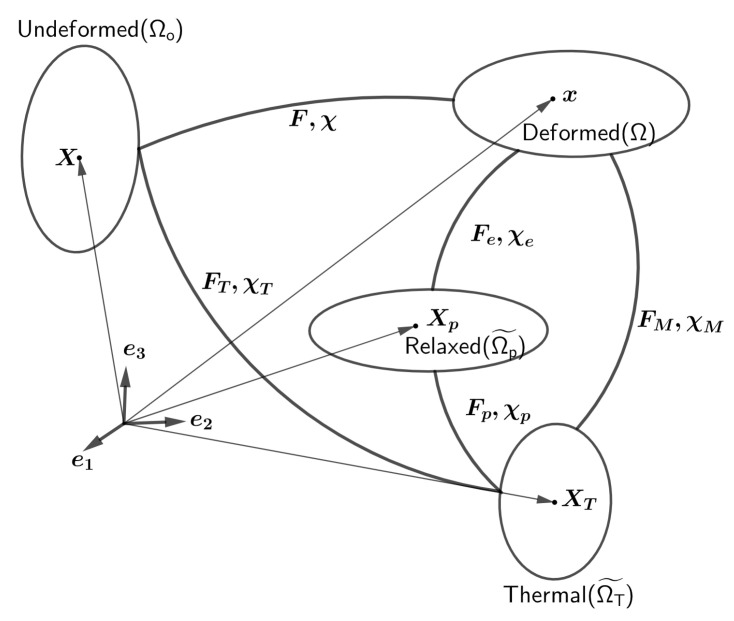
An analogous decomposition scheme for the deformation gradient.

Consider the reference configuration ($\Omega_{o}$) as being in a stress-free state and in thermodynamic equilibrium at temperature $T_{o}$, see Figure 1. A point in the body in the reference configuration ($\Omega_{o}$) is assumed to be at position X. In the case of cold programming, the effect of thermomechanical history should be minimal for an initial state in the glassy phase to be in equilibrium to ensure good predictions. Therefore, the sample should be held for a long period at a temperature sufficiently below $T_{g}$ in the unstressed state that the reference configuration can be assumed to be in equilibrium. Under thermomechanical loading, it undergoes motion χ(X,T(t),t)) and the point originally located at X attains a new spatial position x=χ(X(t),T(t),t) in the deformed configuration (Ω) at temperature T. The two intermediate configurations can be thought of as a thermal configuration (${\tilde{\Omega }}_{T}$) and a stress-relaxed plastic configuration (${\tilde{\Omega }}_{p}$) defined by motions $\chi_{T}$ and $\chi_{p}$, respectively. (${\tilde{\Omega }}_{T}$) is an intermediate configuration of the purely thermally loaded body, while the (${\tilde{\Omega }}_{p}$) configuration is obtained by elastic unloading of the final configuration. The local motion can be decomposed as $\chi =\chi_{M}\left(\chi_{T}\left(X,T\left(t\right),t\right)\right)$. Likewise, the mechanical deformation can be further broken down into elastic and plastic parts, $\chi =\chi_{e}\left(\chi_{p}\left(\chi_{T}\left(X,T\left(t\right),t\right)\right)\right)$.

A convenient measure of deformation is the deformation gradient, which is defined as the material gradient of a spatial position, F=Grad(x). The deformation gradient can be decomposed by a multiplicative splitting of successive motions [39].

(2) $$F=F_{M}F_{T}\;;\;\;\;\;F_{M}=F_{e}F_{p}$$

Equation (3) gives the definition of different strain tensors for different pairs of configurations.

(3) $$F=\frac{\partial \chi }{\partial X};\;F_{T}=\frac{\partial \chi_{T}}{\partial X};\;F_{M}=\frac{\partial \chi_{M}}{\partial X_{T}};\;F_{p}=\frac{\partial \chi_{p}}{\partial X_{T}};\;\;F_{e}=\frac{\partial \chi_{e}}{\partial X_{p}};\;$$

In the case of compressible deformation, the deformation gradient and the strain tensors are often divided into volume-changing (*J*) and volume-preserving ($\bar{F}$) parts [40].

(4) $$F=J^{\frac{1}{3}}\bar{F}\;,\;\;\;J=det\left(F\right)>0$$

where $\bar{F}$ is the deviatoric component of F and *J* is the volume ratio. The deformation due to plastic flow, $F_{p}$, is assumed to be purely distortional; therefore, $F_{p}={\bar{F}}_{p}$. The left Cauchy–Green deformation tensor (b) and right Cauchy–Green deformation tensor (C) for the configurations ${\tilde{\Omega }}_{M}$, ${\tilde{\Omega }}_{e}$, and ${\tilde{\Omega }}_{p}$ together with their volumetric ($J^{\frac{2}{3}}$) and deviatoric ($\bar{C}$ and $\bar{b}$) components are defined as follows:

(5) $$\begin{array}{cc} \; & C_{M}=F_{M}^{T}F_{M}\;,\;b_{M}=F_{M}F_{M}^{T}\;,\;C_{M}=J_{M}^{\frac{2}{3}}{\bar{C}}_{M}\;,\;b_{M}=J_{M}^{\frac{2}{3}}{\bar{b}}_{M}\\ \; & C_{e}=F_{e}^{T}F_{e}\;,\;\;\;b_{e}=F_{e}F_{e}^{T}\;,\;\;\;C_{e}=J_{e}^{\frac{2}{3}}{\bar{C}}_{e}\;,\;\;\;b_{e}=J_{e}^{\frac{2}{3}}{\bar{b}}_{e}\\ \; & C_{p}=F_{p}^{T}F_{p}\;,\;\;b_{p}=F_{p}F_{p}^{T}\;,\;\;C_{p}={\bar{C}}_{p}\;,\;\;b_{p}={\bar{b}}_{p} \end{array}$$

### 3.2. Governing Equations

**Figure 2:**
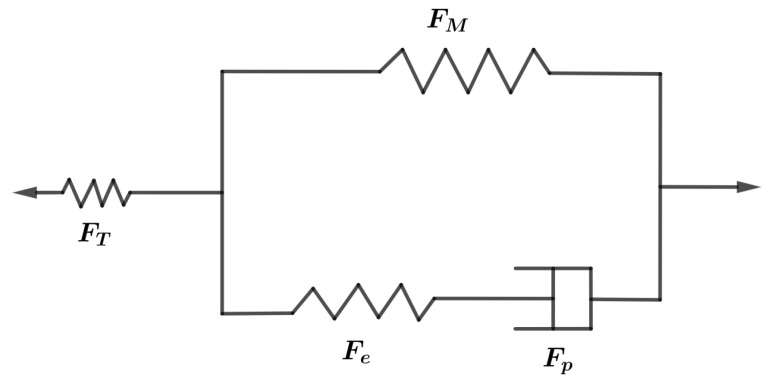
A linear thermoviscoelastic rheological model.

The SMP behaves as an elastic-viscoplastic glassy material at $T<<T_{g}$ and as a hyperelastic rubbery material at $T>>T_{g}$. The rheological model shown in Figure 2 satisfies both the assumptions that $F=F_{M}F_{T}$ and $F_{M}=F_{e}F_{p}$. At $T>>T_{g}$, the dashpot offers no resistance, so only the top branch contributes to the stress. Therefore, the upper branch predicts the hyperelastic and lower branch of the elastic-viscoplastic response.

#### 3.2.1. Mechanical Response

The constitutive equations for the isothermal mechanical response of SMP are derived from the Helmholtz free energy function. The Helmholtz free energy function can be additively split into deviatoric and volumetric parts, which are the functions of devatoric (${\bar{C}}_{e},{\bar{C}}_{p}$) and volumetric ($J_{M}$) components of deformation tensors, respectively. It is convenient to use the objective tensors ${\bar{C}}_{M}$ and ${\bar{C}}_{e}$ by omitting ${\bar{C}}_{p}$.

(6) $$\Psi \left({\bar{C}}_{M},{\bar{C}}_{e},J_{M}\right)=\Psi_{dev}\left({\bar{C}}_{M},{\bar{C}}_{e}\right)+\Psi_{vol}\left(J_{M}\right)$$

The deviatoric component ($\Psi_{dev}$) of the free energy per unit volume is assumed to be the sum of time-independent hyperelastic ($\Psi_{dev}^{h}$) and time-dependent elastic-viscoplastic ($\Psi_{dev}^{ve}$) components.

(7) $$\Psi_{dev}\left({\bar{C}}_{M},{\bar{C}}_{e}\right)=\Psi_{dev}^{h}\left({\bar{C}}_{M}\right)+\Psi_{dev}^{ve}\left({\bar{C}}_{e}\right)$$

In the rubbery phase, the crosslinked chains reorient and align upon deformation. At high temperatures, short-range interactions between molecules disappear, and resistance to deformation is due only to entanglement. Arruda and Boyce (1998b) [41] proposed an eight-network model, which is suitable for predicting the rubbery response undergoing large deformation. The model accounted for eight orientations of chains in space, and the strain energy stored in chains due to deformation is found using the inverse Langevin function. The energy density function is given in Equation (8).

(8) $$\Psi_{dev}^{h}\left({\bar{I}}_{M_{1}}\right)=\mu_{r}\lambda_{L}^{2}\left[\frac{\lambda_{chain}}{\lambda_{L}}\beta +ln\left(\frac{\beta }{sinh\beta }\right)\right]-\Psi_{o},\;\;\;\beta =L^{-1}\left(\frac{\lambda_{chain}}{\lambda_{L}}\right)$$

where $\mu_{r}$ is the shear modulus of the hyperelastic branch, $\lambda_{L}$ is the maximum possible value of chain stretch in the fully extended state known as locking stretch, $\lambda_{chain}$ is the chain stretch, $L^{-1}$ is the inverse Langevin function and $\Psi_{o}$ is a constant. Using Treloar (1976) [42] approximation, the inverse Langevin function $L^{-1}$ can be expressed as:

(9) $$L^{-1}\left(x\right)=\frac{3x}{1-\left(\frac{3}{5}x^{2}+\frac{36}{175}x^{4}+\frac{108}{875}x^{6}\right)}$$

Chain stretch $\lambda_{chain}$ is the ratio of current to initial chain length. It can be simplified in terms of the first principal invariant of mechanical deformation (${\bar{I}}_{M_{1}}$) as follows:

(10) $$\lambda_{chain}=\frac{{\left({\bar{I}}_{M_{1}}\right)}^{\frac{1}{2}}}{\sqrt{3}}$$

In the glassy phase, due to the high viscosity of SMP, the deformation is mainly due to the stretching of linked and unlinked molecules bonded by close-range interaction. The deviatoric component of the strain energy density in the glass phase is modeled using the incompressible Neo–Hookean strain energy density function.

(11) $$\Psi_{dev}^{ve}\left({\bar{I}}_{e_{1}}\right)=\frac{1}{2}\mu_{g}\left({\bar{I}}_{e_{1}}-3\right)$$

where $\mu_{g}$ is the shear modulus of SMP in the glassy phase, and ${\bar{I}}_{e_{1}}$ is the first invariant of the elastic component of mechanical deformation.

The volumetric deformation of SMP is predicted by considering it as a compressible Neo–Hookean material.

(12) $$\Psi_{vol}\left(J_{M}\right)=k\left(J_{M}-lnJ_{M}-1\right)$$

where *k* is the bulk modulus of SMP in the glassy phase and $J_{M}$ is the volume ratio of mechanical deformation.

Considering the Clausius–Plank inequality, the free energy constraint can be imposed as follows:

(13) $$\frac{1}{2}S:{\dot{C}}_{M}-\dot{\Psi }\geq 0$$

where S is the second Piola–Kirchhoff stress tensor. The Cauchy stress can be obtained from the Piola–Kirchhoff stress tensor by transforming it as follows:

(14) $$\sigma =J^{-1}F_{M}SF_{M}^{T}=\sigma_{vol}+\sigma_{dev}^{h}+\sigma_{dev}^{ve}$$

The Cauchy stress (σ) is evaluated from the Clausius–Plank inequality and the strain energy densities from Equations (8)–(14).

(15) $$\begin{array}{cc} \; & \sigma_{vol}=\frac{1}{J}k\left(J_{M}-1\right)I\\ \; & \sigma_{dev}^{h}=\frac{1}{J}\mu_{r}\frac{\lambda_{L}}{\lambda_{chain}}L^{-1}\left(\frac{\lambda_{chain}}{\lambda_{L}}\right)\left({\bar{b}}_{M}-\frac{1}{3}{\bar{I}}_{M_{1}}I\right)\\ \; & \sigma_{dev}^{ve}=\frac{1}{J}\mu_{g}\left({\bar{b}}_{e}-\frac{1}{3}{\bar{I}}_{e_{1}}I\right) \end{array}$$

#### 3.2.2. Glass Transition

To quantify the thermal history of a polymer at a given temperature, Tool (1946) [43] defined an internal variable known as the fictive temperature ($T_{f}$). $T_{f}$ is an imaginary temperature value at which any non-equilibrium glass is assumed to reach equilibrium. For a single mechanism for structural relaxation, the evolution of $T_{f}$ can be written as in Equation (16).

(16) $$\frac{dT_{f}}{dt}=\frac{1}{\tau_{S}}\left(T-T_{f}\right)$$

where $\tau_{S}$ is the structural relaxation time. The initial value of $T_{f}$ is equal to the room temperature; therefore, at t=0 the $T_{f}=T$. For $T<<T_{g}$, $T_{f}$ does not change and is fixed to $T_{g}$. The structural relaxation time depends on the probabilities of cooperative rearrangements of molecule groups [44]. Using this model of structural relaxation, Nguyen (2008) [36] further developed a WLF (Williams–Landel–Ferry)-based form to quantify the structural relaxation time.

(17) $$a_{T}=log\left(\frac{\tau_{S}^{o}\left(T_{g}\right)}{\tau_{S}\left(T\right)}\right)=\frac{C_{1}\left(C_{2}\left(T-T_{f}\right)+T\left(T_{f}-T_{g}\right)\right)}{T(C_{2}+T_{f}-T_{g})}$$

where $a_{T}$ is the shift factor, $\tau_{S}^{o}$ is structural relaxation time at $T_{g}$, $C_{1}$ and $C_{2}$ are first and second WLF constants, and *Q* is the thermal activation energy.

#### 3.2.3. Plastic Deformation

According to the conventional flow rule, the plastic shear strain rate evolves in the direction of flow stress and is inversely proportional to viscosity. The flow rule is given as:

(18) $${\dot{\gamma }}_{p}=\frac{1}{2\eta }∥\sigma_{dev}^{ve}∥$$

where ${\dot{\gamma }}_{p}$ is the plastic shear strain rate, and η is the viscosity of SMP. Flow stress or equivalent shear stress ($\bar{\tau }$) and the direction of flow (n) is given in Equation (19).

(19) $$\begin{array}{cc} \; & \bar{\tau }={\left(\frac{1}{2}\sigma_{dev}^{ve}:\sigma_{dev}^{ve}\right)}^{\frac{1}{2}}\\ \; & n=\frac{\sigma_{dev}^{ve}}{∥\sigma_{dev}^{ve}∥} \end{array}$$

The thermodynamic inequality Equation (13) along with Equation (18) yields the following equation of plastic deformation:

(20) $$-\frac{1}{2}L_{v}b_{e}b_{e}^{-1}={\dot{\gamma }}_{p}\frac{\sigma_{dev}^{ve}}{∥\sigma_{dev}^{ve}∥}$$

where $L_{v}b_{e}$ is the Lie time derivative of $b_{e}$. The flow rule represents the rate of evolution of plastic strain after yielding. The deformation of glassy SMP is non-linear elastic at first. As the strain is increased, the stress builds up due to the resistance to deformation, and after it reaches a certain limit where it overcomes the resistance, plastic flow begins [45]. The plastic shear strain rate of SMP is dependent on temperature and varies through the glass transition zone. The modified Eyring equation, which includes the structural relaxation time for predicting the viscosity change in the glass transition zone, is considered.

(21) $${\dot{\gamma }}_{p}=\frac{s}{\sqrt{2}\eta_{o}}\frac{T}{Q}exp\left(\frac{1}{log\;e}\frac{C_{1}\left(C_{2}\left(T-T_{f}\right)+T\left(T_{f}-T_{g}\right)\right)}{T(C_{2}+T_{f}-T_{g})}\right)sinh\left(\frac{Q}{T}\frac{\bar{\tau }}{s}\right)$$

where $\eta_{o}$ is shear viscosity at reference temperature ($T_{g}$).

On further deformation, the local structure changes such that the shear strength against the rotation of chain segments further decreases; this marks the beginning of strain-softening. Here, the phenomenological softening evolution model of Boyce (1989b) [46] is considered, which takes into account the influence of temperature and strain rate.

(22) $$\dot{s}=h\left(1-\frac{s}{s_{s}}\right){\dot{\gamma }}_{p}\;,\;\;\;s\left(t=0\right)=s_{o}$$

where *s* is the athermal shear strength, $s_{o}$ is the initial value at the upper yield point, $s_{s}$ is the value of *s* at the lower yield point, and *h* is the yield drop slope.

#### 3.2.4. Thermal Response

When a polymer is cooled from temperature $T_{2}$ to $T_{1}$, the property change in *p* is an algebraic sum of the property change from $T_{2}$ to the extrapolated temperature $T_{f}$, and from $T_{f}$ to $T_{1}$ with rates of change as in the rubbery and glassy phase, respectively, refer to Equation (23) [47].

(23) $$p\left(T_{2}\right)-p\left(T_{1}\right)=\left(T_{2}-T_{f}\right){\left(\frac{dp}{dT}\right)}_{T>>T_{g}}+\left(T_{f}-T_{1}\right){\left(\frac{dp}{dT}\right)}_{T<<T_{g}}$$

This property change definition applies to stress-free thermal contraction/expansion that occurs due to temperature changes. Let SMP be initially in thermal equilibrium at $T=T_{o}$ so that volumetric thermal deformation $J_{T}\left(T_{o}\right)=1$. When cooling down to the temperature *T*, the volumetric thermal deformation $J_{T}$ develops according to Equation (24)

(24) $$\begin{array}{cc} J_{T}\left(T\right) & =J_{T}\left(T_{o}\right)-\alpha_{r}\left(T_{o}-T_{f}\right)-\alpha_{g}\left(T_{f}-T\right)\\ \; & =1-\alpha_{r}\left(T_{o}-T_{f}\right)-\alpha_{g}\left(T_{f}-T\right) \end{array}$$

where $\alpha_{g}$ and $\alpha_{r}$ are volumetric coefficients of thermal expansion in the glassy and rubbery phase, respectively.

## 4. Numerical Implementation

Uniaxial tensile behavior is simulated and validated experimentally. For the sake of simplicity, the tensor equations have been converted to algebraic equations in Appendix A. The differential equations required to solve the loading, stress-relaxation, unloading, isothermal recovery, and heated recovery steps are derived in Appendix B. The material parameters are determined through unique experiments. Some parameters are measured directly from experiments, while some required curve fitting, as discussed in Section 5.1. The material constants are then used to validate the simulations and predict the response of SMP at different temperatures and stress relaxation times.

## 5. Results and Discussion

### 5.1. Material Parameters of Proposed SMP

The material parameters are determined through unique experiments and through simulation results. This is performed in such a way that we encounter a minimal number of unknown material parameters at startup and use their values in the next step to simulate behavior that requires more unknown parameters. Some parameters were measured directly from experiments, while some required an analysis. The material parameters are summarized in Table 1.

**Figure 3:**
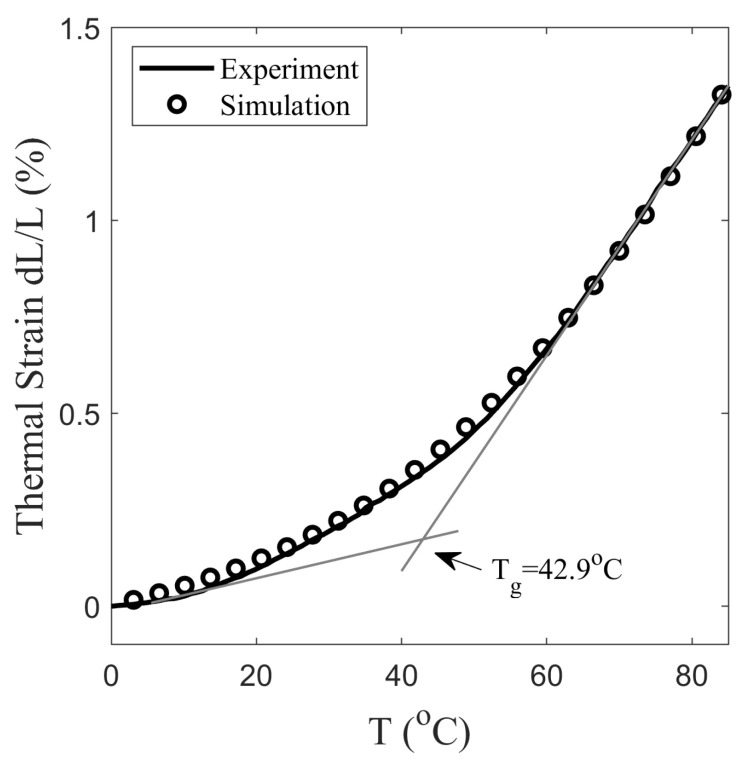
Evolution of thermal strain measured by DMA of SMP subjected to stress-free cooling at 4 °C/min.

**Figure 4:**
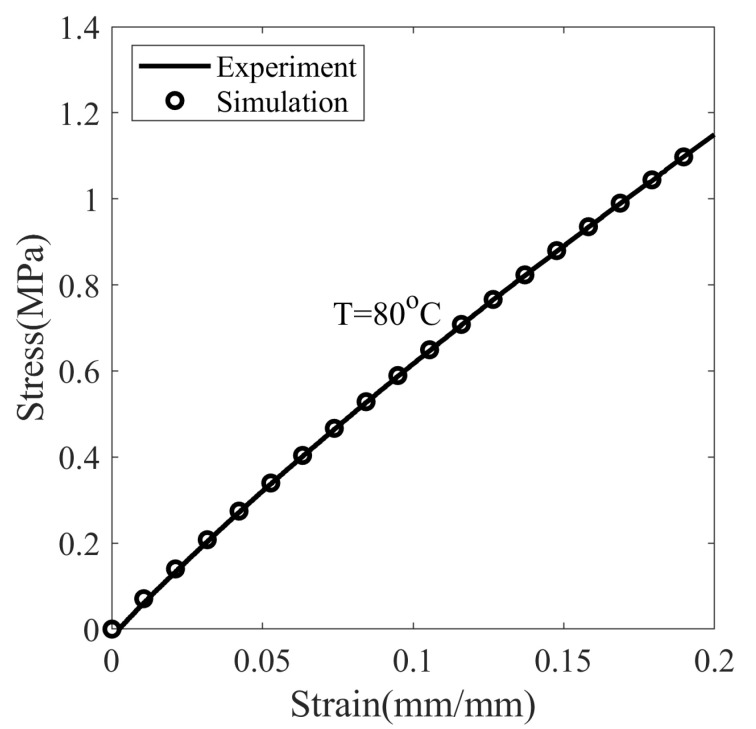
The stress–strain response of SMP deformed with a strain rate of 0.0013 s${}^{-1}$ at T = 80 °C in the rubbery state.

First, the material parameters of the glass transition are determined from the stress-free cooling curve of SMP (Figure 3). The slopes of the curve at 10 and 80 °C are 4.44 × 10${}^{-5}$ and 2.79 × 10${}^{-4}$, and the temperature at the intersection of the tangents is 42.9 °C. By considering the slope and intersection, the values are $\alpha_{g}=$ 1.32 × 10${}^{-4}$ °C${}^{-1}$, $\alpha_{r}=$ 8.38 × 10${}^{-4}$ °C${}^{-1}$, and $T_{g}=$ 42.9 °C. The Equation (24) is solved with the initial condition of $T_{f}=T$ at t=0 to simulate thermal strain. From simulations and the values of experiment, $C_{1}$ = 19.1, $C_{2}$ = 58.0 °C, and $\tau_{S}^{o}$ = 1573 s are obtained. The hyperelastic parameters are determined from the nominal stress–strain curve at T =80 °C deformed with a strain rate of 0.0013 s${}^{-1}$, see Figure 4. The value of Cauchy stress $\sigma_{dev}^{h}$ given in Equation (15) is converted to the Piola–Kirchhoff stress $S_{dev}^{h}$ using Equation (14) and matched with the experiment for determining $\lambda_{L}$ and $\mu_{r}$. With the assumption, $\lambda_{chain}=1$ resulted in $\lambda_{L}=$ 1.6 and $\mu_{r}=$ 0.9 MPa.

**Figure 5:**
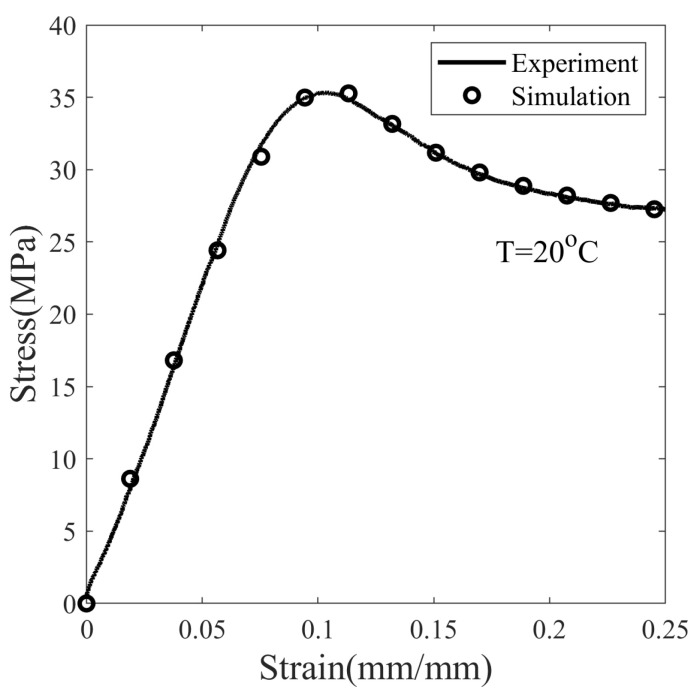
The stress–strain response of SMP deformed with a strain rate of 0.0013 s${}^{-1}$ at T = 20 °C in the glassy state.

**Figure 6:**
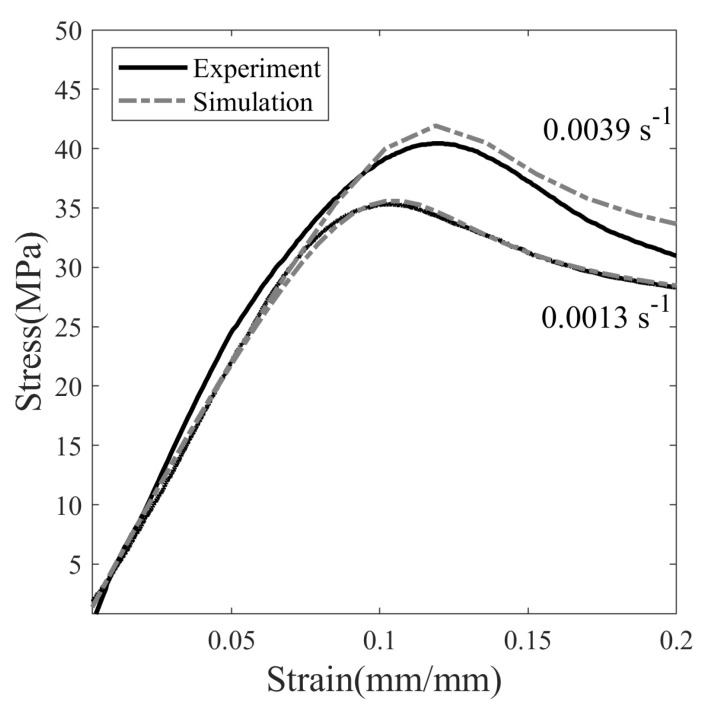
The stress–strain response of deformed SMP at strain rates of 0.0013 and 0.0039 s${}^{-1}$.

The glassy elastic modulus (*E*) is measured from the initial slope of the nominal stress–strain curve at T =20 °C and deformed at a strain rate of 0.0013 s${}^{-1}$, see Figure 5. The Poisson’s ratio in the glassy state of SMP is taken as ν= 0.36 [36]. Therefore, the elastic constants measured at T =20 °C are E= 459.1 MPa, $\mu_{g}=$ 168.9, and *k* = 543.1 MPa. The viscoelastic parameters are determined from the stress–strain curve at different strain rates. Figure 6 shows the stress response of SMP at 20 °C deformed at strain rates of 0.0013 s${}^{-1}$ and 0.0039 s${}^{-1}$. The nominal stress S in Equation (14) is fitted with the experimental result shown in Figure 5 and Figure 6. This results in $s_{o}$ = 20.017 MPa, $\eta_{o}=$ 1.48 ×$10^{4}$ MPa-s, $s_{s}/s_{o}=$ 0.7, $Q/s_{o}=$ 72.0 K/MPa, and h= 300.0 MPa.

### 5.2. Experiments and Model Validation

#### 5.2.1. Glass Transition

**Figure 7:**
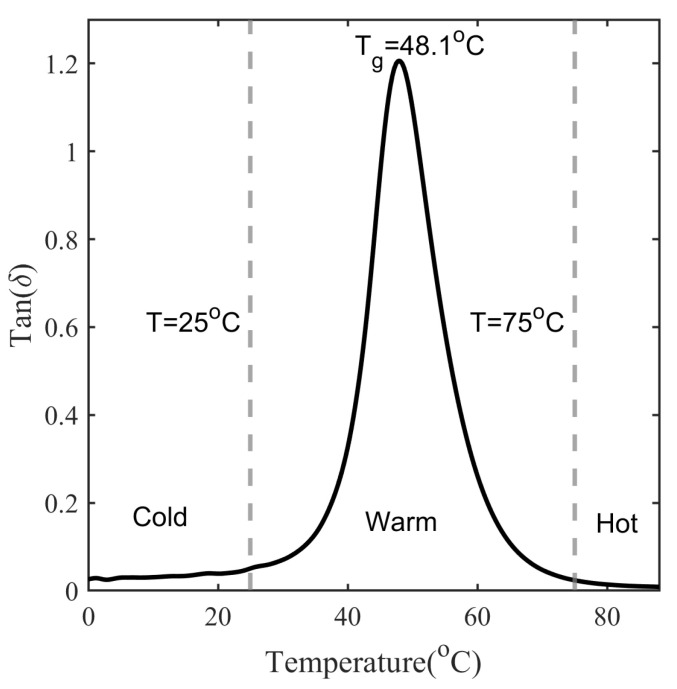
Tan δ measured with DMA at a frequency of 1 Hz and a heating rate of 4 °C/min.

The loss factor (tan δ) generally describes the damping behavior of a material. Figure 7 shows the loss factor of SMP measured with DMA at a frequency of 1 Hz and a heating rate of 4 °C/min. The inflection points at the onset and end of the glass transition divide the temperature axis into cold, warm and hot programming zones [21]. In this case, the glass transition of SMP begins almost at 25 °C and ends at 75 °C, peaking at 48 °C. Therefore, if the programming temperature of the SMP is below 25 °C, it can be referred to as cold programming, from 25 and 75 °C warm programming and above 75 °C hot programming. The temperature at the peak of the tan δ curve is referred to as the glass transition temperature. However, the glass transition temperature measured from the thermal contraction curve is accounted for in the analytical model, as discussed in Section 5.2.2.

#### 5.2.2. Structural Relaxation

The thermal strain of the stress-free cooled SMP is measured with DMA, as described in Section 2.2. Figure 3 shows that thermal strain does not vary linearly with temperature. The nonlinearity arises due to a time-dependent non-equilibrium structural relaxation that takes place during the phase change. On cooling the SMP from a temperature T=90 °C to 0 °C at 2 °C/min, it shrunk with 1.3% strain as it transitioned from the rubbery state to the glassy state. The glass transition temperature is measured in Figure 3, which is the temperature at the intersection of the extrapolation tangent at $T<T_{g}$ and $T>T_{g}$ of the strain curve. It corresponds to an equilibrium state in which the structural relaxation stops and the fictive temperature $T_{f}$ approaches $T_{g}$ [47]. The $T_{g}$ is measured as 42.9 °C at the intersection of tangents at T=10 °C and 80 °C. In addition, the slope of the tangents gives the linear thermal expansion coefficients, which can be approximated to about one-third of the volumetric thermal expansion coefficients ($\alpha_{g}$ and $\alpha_{r}$). The measured values of $\alpha_{g}$, and $\alpha_{r}$ are 1.32 × $10^{-4}$ and 8.38 × $10^{-4}$ °C${}^{-1}$, respectively. Equations (16) and (24) are solved to obtain $J_{T}$, which is used to calculate the thermal strain in Figure 3. The temperature is changed from T=90 °C to 0 °C at 4 °C/min and the initial value of $T_{f}$ is considered 90 °C for the simulation. The analysis of experiments gives the structure relaxation time at the glass transition temperature of 1573 s.

#### 5.2.3. Stress Response

**Figure 8:**
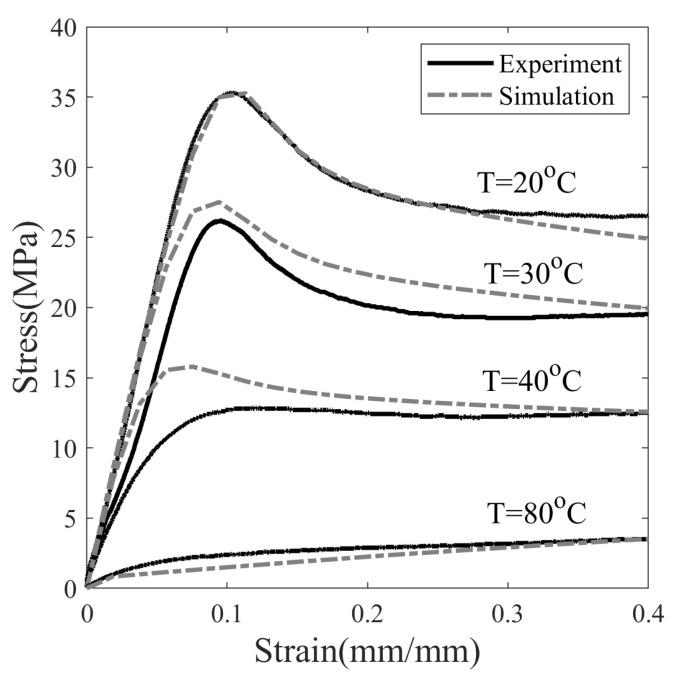
Stress response of uniaxial tension at a strain rate of 0.0013 s${}^{-1}$ of SMP at different temperatures.

Figure 8 shows the nominal stress–strain response of SMP at different temperatures, deformed with a constant strain rate of 0.0013 s${}^{-1}$. Based on observations, it is assumed that the material behaves in a linear-elastic manner at a small strain value (approx 0.01). The modulus of elasticity is measured in this linear range. It is observed that the elastic modulus dropped drastically from 325.89 to 6.08 MPa as the temperature increased from 20 to 80 °C. The chain mobility of SMP increases with increasing temperature due to the decrease in short-range intermolecular and intramolecular interaction. As a result, SMP builds up low stresses at elevated temperatures and thus reduces rigidity. When the temperature of SMP is sufficiently above $T_{g}$ (T = 80 °C), short-range interactions disappear completely, and the deformation resistance is entirely due to permanent crosslinked bonds; hence, the stress keeps increasing, as shown in Figure 8.

At 20 °C, the SMP showed yielding when the strain level is increased to 10%. The distinct point where the stress starts to decrease has been marked as the upper yield point of the SMP. SMP yielding occurs when the stress developed is large enough to cause segmental rotations. However, no distinct yielding is observed at a temperature of 40 °C, which is close to $T_{g}$. As in Figure 8, the yield strengths of SMP at 20 and 30 °C are 35.28 and 26.21 MPa, respectively. After SMP has reached the yield point, the local structure changes in such a way that the shear resistance to rotation of the chain segments further decrease [45]. Above a strain value of 10% for SMP at 20 °C, strain-softening is observed, followed by steady plastic flow at a stress of 25.30 MPa, also known as saturation yield strength. The ratio of saturation to the upper yield point is about 0.7. The strain zone after the onset of yielding is considered appropriate for cold programming because plastic deformation is required to impart non-recoverable plastic deformation to SMP at ambient temperature.

The model is validated by simulating the stress response at different temperatures, see Figure 8. The set of differential equations in Appendix B.1 is solved simultaneously with the initial conditions in Appendix B.2 to predict stress response. The maximum value of the strain is assumed to be 40% and the strain rate to be 0.0013 s${}^{-1}$. At t=0, it is assumed that SMP is undeformed and in equilibrium. There is a good agreement between experimental and simulated values for stress, yield strength, strain-softening observed at 20 (cold zone) and 80 °C (rubbery zone), and deterioration in fit is observed in the warm zone as the temperature increased from 30 to 40 °C. A possible way to mitigate this deviation is to use temperature-dependent material properties. Since, as in Figure 8, the drop in the modulus of elasticity as the temperature increases is not captured with the current model, the gap between the experimental and the simulated value increases with the onset of the deformation.

#### 5.2.4. Thermomechanical Cycle

**Figure 9:**
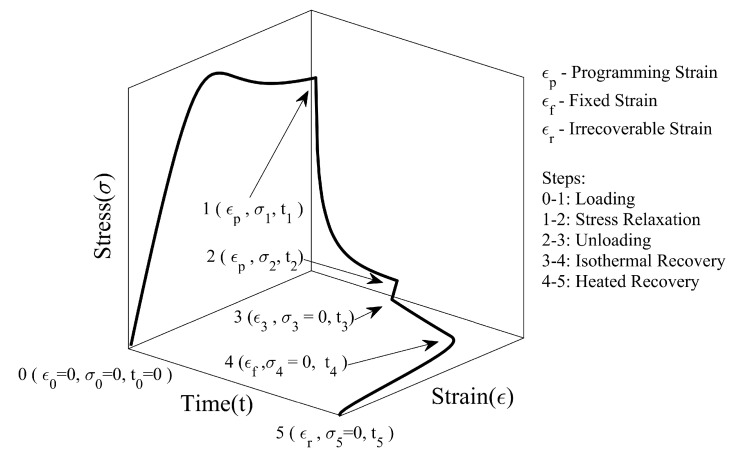
Representation of steps involved in the thermomechanical cycle in cold programming of SMP.

Figure 9 shows the stress–strain–time response of SMP undergoing a complete thermomechanical cycle. In the first step (0→1), the SMP is deformed at room temperature until it reaches the desired programming strain ($\epsilon_{p}$). We prefer to use programming temperature ($T_{p}$) terminology rather than room temperature, as we have considered various room temperatures for the studies. After the loading step, the stress is allowed to relax from $\sigma_{1}$ to $\sigma_{2}$ for a period of $t_{r}=t_{2}-t_{1}$, keeping the strain constant (1→2). When the material is unloaded (2→3), the stress immediately drops to zero, and spring-back recovery of strain ($\epsilon_{p}-\epsilon_{3}$) occurs. In the fourth step (3→4), the SMP is maintained in a stress-free state at temperature $T_{p}$ to study the effect of isothermal viscoelastic recovery on shape fixity. The shape fixity ($S_{f}$) is defined as the ratio of fixed strain ($\epsilon_{f}$) after the shape is fixed to maximum strain ($\epsilon_{p}$) imparted during deformation, see Equation (25).

(25) $$S_{f}=\frac{\epsilon_{f}}{\epsilon_{p}}$$

where $S_{f}$ is shape fixity, $\epsilon_{f}$ is fixed strain, and $\epsilon_{p}$ is programming strain. In the present work, shape fixity is split into instantaneous and gradual parts. It is defined as follows:

(26) $$S_{f}\equiv \frac{\epsilon_{3}}{\epsilon_{p}}\frac{\epsilon_{f}}{\epsilon_{3}}=S_{f}^{i}S_{f}^{g}$$

where $S_{f}^{i}$ is instantaneous shape fixity, and $S_{f}^{g}$ is gradual shape fixity.

The last step (4→5) is to heat SMP above $T_{g}$, where SMP reverts from its temporary form to its original form. In certain cases, even after heating the SMP, some amount of irrecoverable strain ($\epsilon_{r}$) may remain. To characterize the shape regaining ability, the recovery ratio ($R_{r}$) is defined as the ratio of the recovered strain($\epsilon_{f}-\epsilon_{r}$) to fixed strain ($\epsilon_{f}$).

(27) $$R_{r}=\frac{\epsilon_{f}-\epsilon_{r}}{\epsilon_{f}}$$

where $R_{r}$ is the recovery ratio, $\epsilon_{f}$ is the fixed strain, and $\epsilon_{r}$ is a non-recoverable or residual strain.

#### 5.2.5. Effect of Programming Temperature

**Figure 10:**
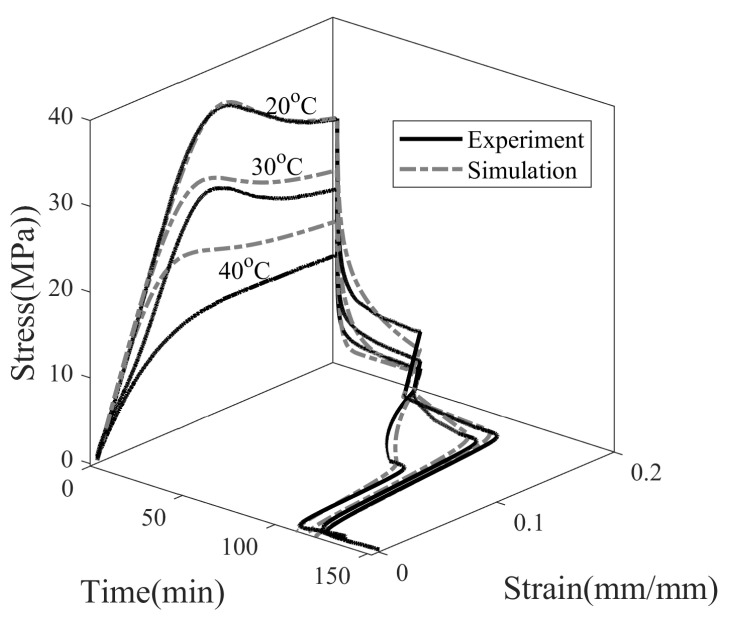
Thermomechanical behavior of SMP at programming temperatures, 20, 30, and 40 °C (with $\epsilon_{p}=$ 0.2, $t_{r}=$ 45 min).

Shape fixity not only depends on the material property but also on programming parameters. It can be influenced by the programming temperature ($T_{p}$), the programming strain ($\epsilon_{p}$), and the stress relaxation time ($t_{2}-t_{1}$). In the experiments, the programming strain is kept constant at 20%, and other programming variables are varied to study the effect on shape memory properties. It can be seen in Figure 8 that the stress value in SMP exceeds yield strength and strain softening beyond 20% strain, and it is reasonable enough to consider it a programming strain. To understand the effect of $T_{p}$, three temperatures are considered, i.e., namely 20, 30, and 40 °C, Figure 10. The choice of $T_{p}$ is made such that the effect of temperatures both near and far from $T_{g}=42.9$ °C on shape fixity can be studied.

Figure 10 shows the experimental and simulation results of the full thermomechanical response of SMP programmed at 20, 30, and 40 °C. SMP is deformed at 0.0013 s${}^{-1}$ to a maximum strain of 20%, and the stress is relaxed for 45 min. Next, it is unloaded, during which stress dropped to zero and SMP exhibited an instantaneous spring back. A new shape of SMP is attained; however, SMP is observed to gradually recover on its own with no change in ambient temperature. This step is called isothermal recovery. Due to time constraints, isothermal recovery is observed for 45 min. In the last step, the temperature of the SMP is increased from $T_{p}$ with 4 °C/min to 80 °C. The SMP resumed its original shape, and the plastic deformation is reversed upon heating. This step is called the shape recovery step.

**Figure 11:**
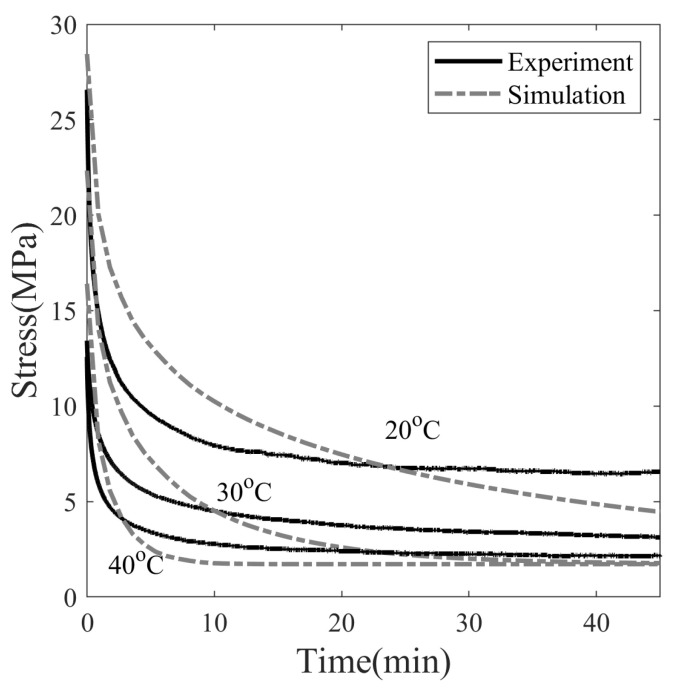
Effect of programming temperature on stress relaxation of SMP at 20% pre-strain.

The programming temperature influences the stress–relaxation behavior of SMP, as shown in Figure 11. The final stress values after 45 min of relaxation are 6.56, 3.12 and 2.14 MPa at temperatures of 20, 30, and 40 °C, respectively. Therefore, stress decays slowly at lower temperatures and rapidly at higher temperatures. The simulation results of 30 and 40 °C show that the stress relaxed to 1.77 MPa within 45 min and asymptotically with the time axis, while the stress of SMP deformed at 20 °C is not able to fully relax in the observed span of time. The dependence of the stress relaxation time of SMP on the programming temperature can be attributed to the dependence of viscosity on temperature. The greater the viscosity, the longer the relaxation time, and vice versa.

As in Figure 2, the dashpot next to the stretched elastic spring in the Maxwell element allows the spring to relax during the stress relaxation process. Both the viscosity of the dashpot and the stiffness of the non-equilibrium spring determine the stress relaxation time. The stiffness of the dashpot is higher at lower temperatures, which causes the stress to relax slowly. To predict stress relaxation more accurately, multiple non-equilibrium mechanisms should be considered; however, only a single mechanism is considered to limit the number of model parameters.

**Figure 12:**
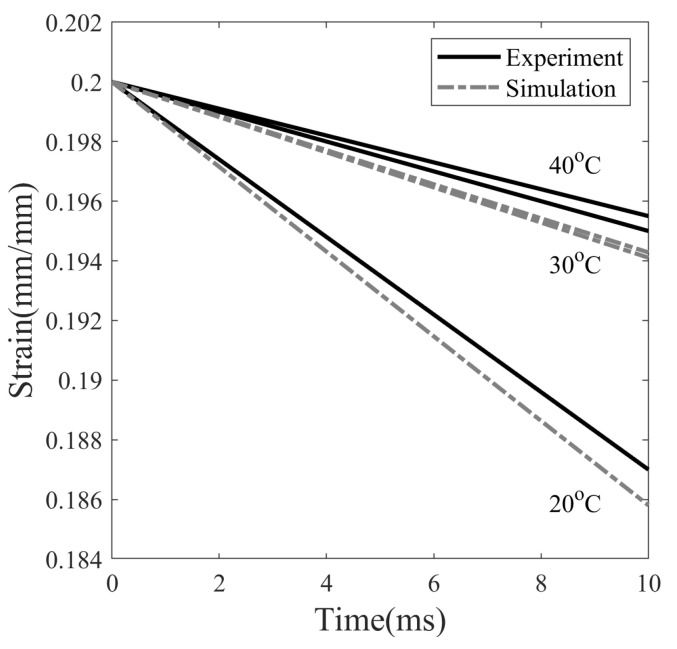
Instantaneous strain recovery of SMP at three different programming temperatures: 20, 30, and 40 °C due to unloading.

The decrease in the strain value when unloading the SMP at different temperatures is plotted in Figure 12. The instantaneous strain decrease is found to be 6.5%, 2.5%, and 2.25% at temperatures of 20, 30 and 40 °C, respectively. Experimental and simulation data showed a similar trend of greater drop in strain values for SMP at 20 °C compared to 40 °C. It is observed that the springback is greater when the stress is higher at the end of the stress relaxation step in SMP, see Figure 12. The shape fixity, evaluated according to Equation (25) after the unloading step, is 93.5%, 97.5%, and 97.75%, with stress values at the end of stress relaxation at 6.56, 3.12, and 2.14 MPa, respectively. Simply put, the higher the stored elastic energy prior to the unloading step, the greater the elastic recovery upon unloading.

**Figure 13:**
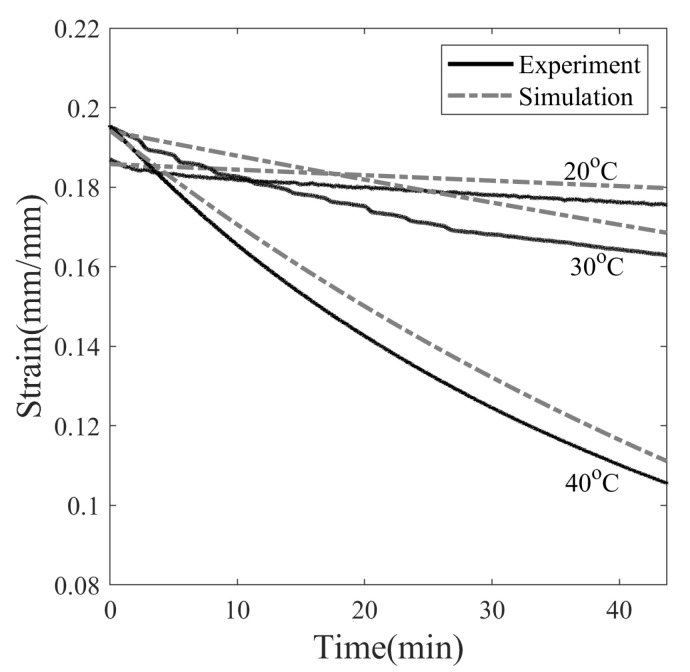
Stress-free isothermal recovery of SMP at temperatures 20, 30, and 40 °C after fixation of the temporary shape.

The ambient temperature around the SMP during operation or storage can change depending on the external factors; therefore, it is important to consider the viscoelastic recovery step for practical applications. Here, the programming temperature of 20 °C is in the cold zone while 30 and 40 °C are in the warm zone. It can be seen in Figure 13 that SMP tends to recover by itself after the unload step without any stimulus. The values of strains after the 45 min of isothermal recovery are 0.175, 0.162, and 0.105 for SMPs programmed at temperatures of 20, 30, and 40 °C, respectively. The shape fixity of SMP programmed at 40 °C drops from 97.75% to 52.75% in 45 min under stress-free and isothermal conditions. Similarly, the shape fixity decreases from 97.35% to 81.15% and from 93.5% to 87.8% for SMPs programmed at 30 and 20 °C, respectively. Therefore, over time, the SMP shrinks rapidly when programmed at 40 °C while shrinking very slowly for those programmed at 20 °C. From the above results, it can be concluded that the isothermal recovery is higher in the glass transition zone and lower in the glassy state. Shape recovery in the glass state relies on a viscoelastic response, while shape recovery within the glass transition zone relies on structural relaxation. Structure relaxation occurs faster during heating. This behavior can be attributed to the reduced viscosity of SMP at higher temperatures, slowly relaxing the frozen structure and reversing some degree of plastic strain in the observed time frame.

**Figure 14:**
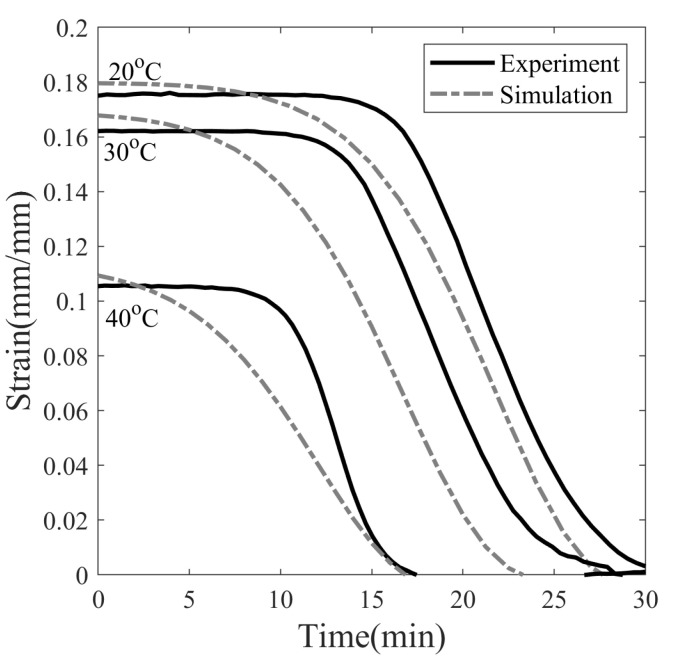
Shape recovery of SMP on heating at 4 °C/min after the isothermal recovery step at three different programming temperatures: 20, 30, and 40 °C.

In the present work, shape recovery of SMP is achieved using heat as a stimulus. SMPs are heated from $T_{p}$ to 80 °C at 4 °C/min in a closed chamber. It can be seen in Figure 14 that the strain value of SMP after heating over $T_{g}$ decreased from 0.175, 0.162 and 0.105 to 0 for all cases. Therefore, 100% shape recovery is observed for SMPs programmed at 20, 30, and 40 °C. The temperature rise inside the SMP sample takes longer than the ambient temperature due to the time-dependent thermal conduction inside the sample during heating. The heat recovery simulation does not take heat conduction into account, so the onset of recovery in the simulation occurred earlier than in the experiment, see Figure 14.

#### 5.2.6. Effect of Stress Relaxation Time

**Figure 15:**
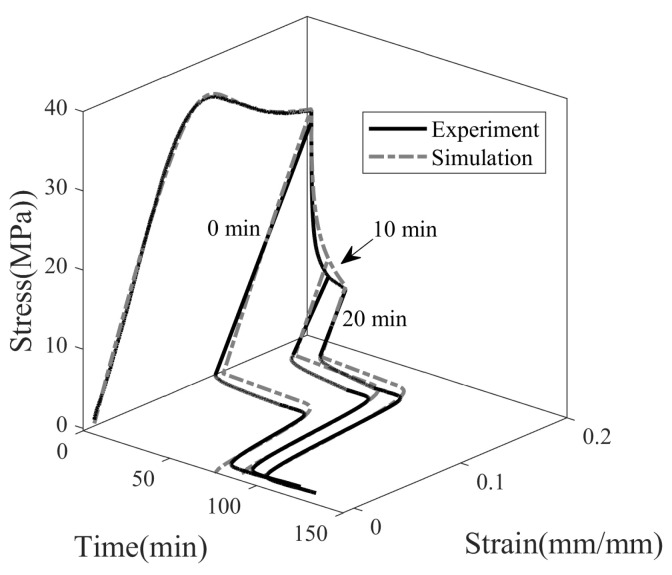
Thermomechanical behaviour of SMP at three different stress relaxation times: 0, 10, and 20 min (with $\epsilon_{p}=$ 0.2, $T_{p}=$ 20 °C).

**Figure 16:**
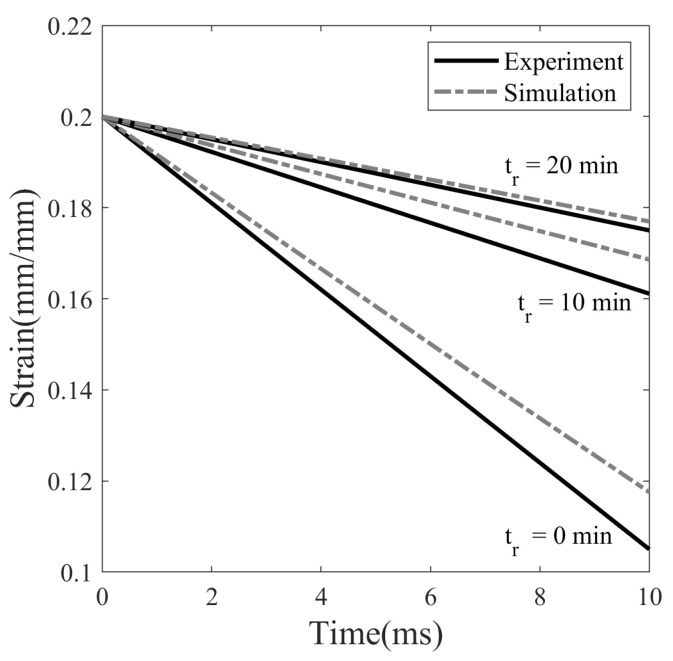
Instantaneous strain recovery on unloading for three different cases of stress relaxation times: 0, 10, and 20 min.

To study the effect of stress relaxation time on shape fixity, three different thermomechanical cycles are performed, as shown in Figure 15 at $T_{p}=20$ °C with different stress relaxation times, i.e., 0, 10, and 20 min. The SMP is deformed to 20% strain at a strain rate of 0.0013 s${}^{-1}$, followed by stress-relaxation. It is then unloaded and observed for isothermal recovery over 45 min. Finally, the SMP is heated from 20 to 80 °C at 4 °C/min to bring it back to its original shape. It can be seen in Figure 15 that stress relaxation is a crucial step in fixing a temporary shape of SMP with the cold programming method. The unloading step in Figure 15 is shown separately in Figure 16, which depicts the immediate recovery of SMPs that are stress-relaxed for the different time periods. Strain decreased from 20% to 11.0%, 16.7%, and 17.6% for cases with a stress relaxation time of 0, 10, and 20 min, respectively. The value of strain after unloading in the case with 0 min stress relaxation time represents the minimum value of plastic deformation that can be obtained in the cold drawing. The shape fixity calculated from the strain value is 55% for a relaxation time of 0 min. This shows that the temporary form of SMP is poorly fixed without stress relaxation. When the relaxation time is increased from 0 to 10 and 20 min, the shape fixity improved from 55% to 83.5% and 88%, respectively. However, as shown in Figure 15, stress relaxation time does not affect isothermal recovery and heated recovery of SMP.

### 5.3. Predictions

#### 5.3.1. Optimum Programming Temperature

**Figure 17:**
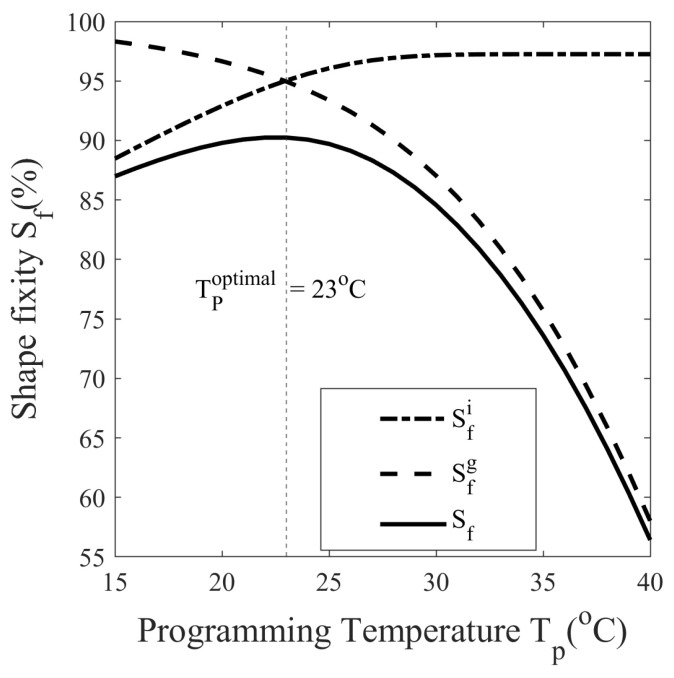
Variation of instantaneous, gradual, and overall shape fixity measured after the isothermal recovery step of thermomechanical cycles performed at programming temperatures ranging from 15 to 40 °C.

The experiments performed in Section 5.2.4 are not sufficient to study the shape memory behavior of SMP programmed with different conditions. Numerous thermomechanical cycles must be run at close intervals of programming temperatures ($T_{p}$), programming strains ($\epsilon_{p}$), and stress relaxation times ($t_{r}$) to understand their effect on shape fixity. To avoid such labor-intensive and costly experiments, simulations are performed. In Figure 17, the dependence of the shape fixity on the programming temperature is plotted. To achieve this, 26 different thermomechanical cycles are simulated with programming temperatures ranging from 15 to 40 °C at an interval of 1 °C, with other variables held constant. The programming strain, stress relaxation time, and isothermal recovery time for each cycle are set at 20%, 45 min, and 45 min, respectively. At $T_{p}=20$ °C and 27 °C, the shape fixity is 89.79% and 88.34%, indicating that SMP is suitable for room temperature applications, see Figure 17.

As in Figure 17, $S_{f}^{i}$ increases from 88.47% to 97.25%, while $S_{f}^{g}$ decreases from 98.32% to 58.01% as the programming temperature increases from 15 to 40 °C. This behavior is also shown in Figure 12 and Figure 13, in that when the programming temperature is high, the instantaneous recovery is less while the isothermal recovery is high. At higher temperatures, the SMP is less stiff, resulting in lower springback recovery and, therefore, increased shape fixity when instantaneously unloaded, while SMP’s low viscosity results in rapid isothermal recovery due to structural relaxation that reduces shape fixity. A peak is observed at $T_{p}=23$ °C in the shape fixity versus programming temperature curve, as shown in Figure 17. This peak indicates that it is possible to reach a maximum shape fixity of 90.24% when the programming temperature is chosen as $T_{p}=23$ °C for SMP programmed at 20% strain and 45 min stress relaxation time. This temperature is referred to as the optimal programming temperature ($T_{p}^{optimal}$) at a given programming strain and stress relaxation time. Between $T_{p}=20$ °C and 27 °C, the shape fixity is 89.79% and 88.34%, which suggests that SMP is suitable for room temperature applications.

**Figure 18:**
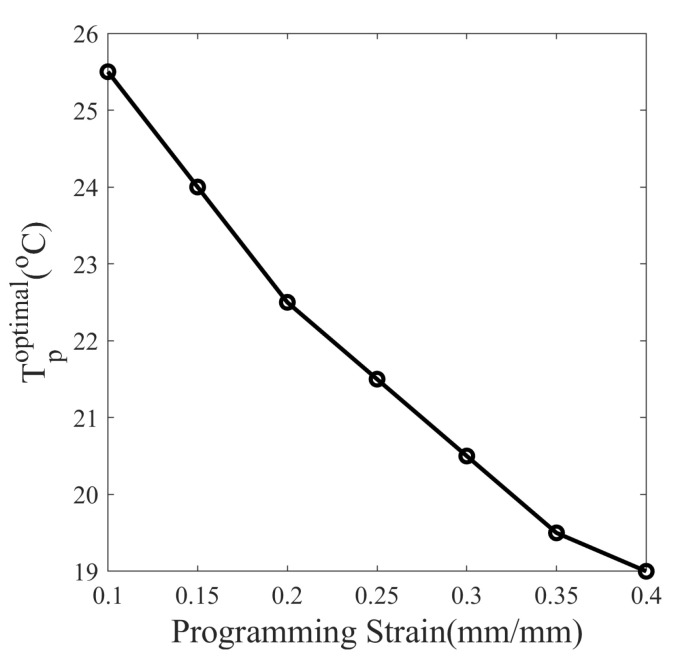
Dependence of optimum programming temperature ($T_{p}^{optimal}$) on programming strain of the SMP.

Next, the programming strain is also varied from 10% to 40% in a 5% interval along with the programming temperature from 15 to 40 °C, resulting in 182 different thermomechanical cycles. The stress relaxation time is set at 45 min for each cycle. The optimal temperature for maximum shape fixity corresponding to each programmed strain is plotted in Figure 18. It is observed that the optimal programming temperature for maximum shape fixity decreases from 25.5 to 19 °C as programming strain increases from 10% to 40%.

The shape fixity is dependent on material properties and programming conditions as well. The following equation gives an expression for optimum temperature for maximum shape fixity shown in Figure 17 and Figure 18:

(28) $${\left.\frac{\partial S_{f}\left(\phi_{SMP},\epsilon_{p},T_{p},t_{r}\right)}{\partial T_{p}}\right|}_{T_{p}=T_{p}^{optimal}}=0$$

where $\phi_{SMP}$ is the material properties of SMP, $\epsilon_{p}$ is the programming strain, $T_{p}$ is programming temperature, and $t_{r}$ is stress relaxation time.

It is important to understand the variation of shape fixity with programming temperature changing from the cold programming zone to the warm programming zone for SMP with $T_{g}$ in the vicinity of room temperature; refer to Figure 7 and Figure 17. It is possible that due to the increase in atmospheric temperature, the SMP, originally designed to follow cold programming in the glassy state at room temperature, has now entered the glass transition. To avoid this situation, the atmospheric temperature should be below $T_{p}^{optimal}$:

(29) $$T_{p}\leq T_{p}^{optimal}$$

From a design point of view, the $T_{g}$ should be tailored such that $T_{p}^{optimal}\geq T_{p}$. The $T_{g}$ can be altered by changing the crosslinking density of the polymer.

#### 5.3.2. Maximum Stress Relaxation Time

**Figure 19:**
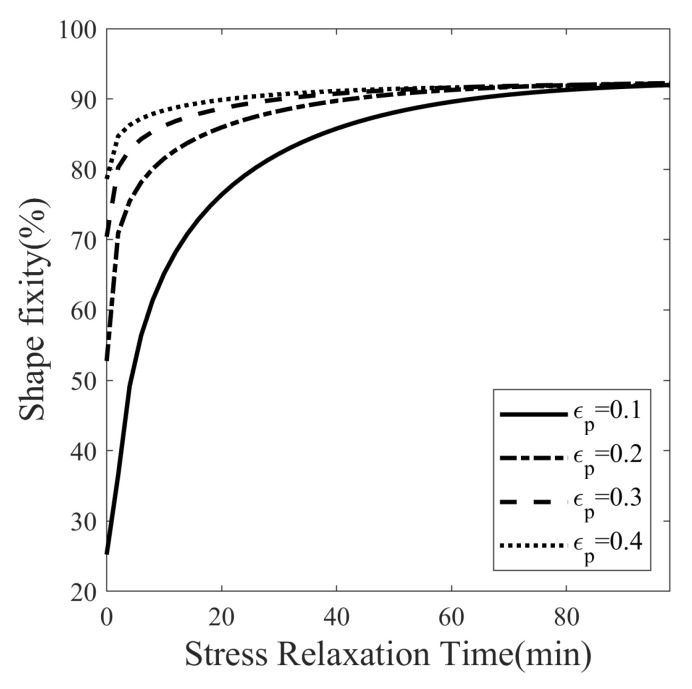
Effect of the stress relaxation time on the shape fixity of SMP at different programming strains ($\epsilon_{p}$).

The effect of stress relaxation time on shape fixity is shown in Figure 19. The programming temperature is set at 23 °C, and four values of programming strain are considered: 10%, 20%, 30%, and 40%, respectively. The stress relaxation time for each case above is varied from 0 to 100 min at narrow intervals of 2 min. Figure 19 shows the shape fixity from a total of 200 thermomechanical cycles. The shape fixity of SMPs programmed at 10%, 20%, 30%, and 40% strain with no stress relaxation is 25.23%, 52.74%, 70.41%, and 78.61%, respectively. The shape fixity saturated to the value of 92.25% for more than 100 min for the stress-relaxed SMPs. Therefore, the maximum attainable shape fixity for the SMP is 92.25%. It means that if the stress relaxation time is increased, there is no further room for improvement in shape fixity. Furthermore, the saturated shape fixity is the same for different programming strains.

**Figure 20:**
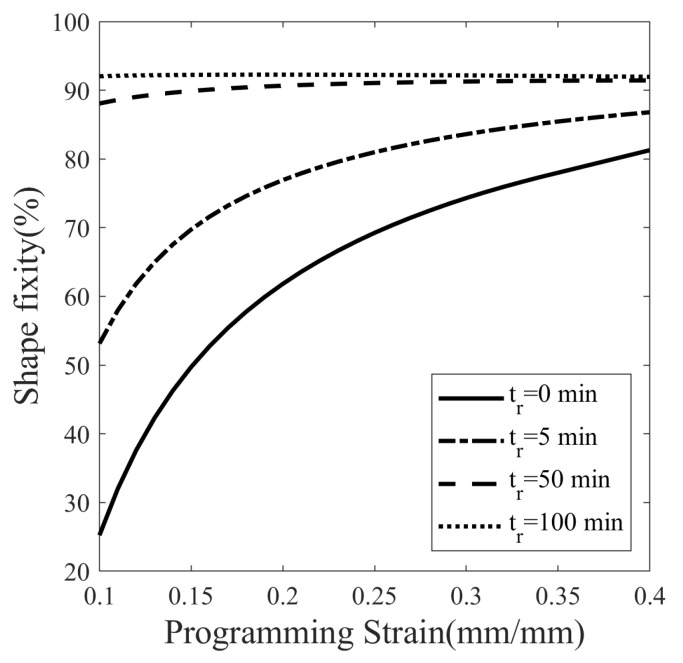
Effect of the programming strain on the shape fixation of SMP at different stress relaxation times ($t_{r}$).

To understand the continuous variation in shape fixity, another case study is conducted. The programming load is varied from 10% to 40%, with an increment of 1% for each value of stress relaxation time, 0, 5, 50, and 100 min. A total of 124 thermomechanical cycles are simulated, and the deflection temperature of each cycle is plotted in Figure 20. The aforementioned saturated shape fixity can be seen as a horizontal line in the graph corresponding to 100 min stress relaxation in Figure 20. However, in cases with a short stress relaxation period, shape fixity increased rapidly with programming strain. It can be observed that the shape fixity increases rapidly from 87.04% to 91.29% for a 0 min relaxation curve while slowly increasing from 88.07% to 91.95% for a 50 min relaxation curve. Therefore, the rate of increase in shape fixity is higher when the programming strain is small and gradually becomes smaller as the programming strain increases.

## 6. Conclusions

An SMP is synthesized using the polymers DGEBA:NGDE in a proportion of approximately 13:7 such that it can be programmed at room temperature. To exhibit large deformation, the $T_{g}$ of SMP is lowered using NGDE and brought sufficiently close to room temperature. The SMP shows an elasto-viscoplastic nature in the glassy state at 20 °C with a clear yield point at 35.28 MPa and a hyperelastic nature in the rubbery state at 80 °C. The mechanical and thermal properties change significantly in the glass transition zone. The $T_{g}$ measured by DMA in creep mode is 42.9 °C, while in dynamic mode, it is 48.1 °C. The strain measured during thermal contraction is non-linear in nature due to the occurrence of time-dependent structural relaxation. Cold programming has advantages over hot programming because it does not require heating to deform and cooling to fix the shape. A five-step thermomechanical cycle, including loading, stress relaxation, unloading (spring back), isothermal recovery (stress-free), and heated recovery, is considered to study the effect of temperature on shape fixity. The isothermal recovery caused a major deterioration in the shape fixity of the SMP. Without a cooling step after loading, the SMP showed a shape fixity of 87.80% at 20 °C (cold zone) while 52.75% at 40 °C (warm zone) over a period of 45 min. The result shows that shape fixity is dependent on the vicinity of $T_{p}$ to $T_{g}$. Since springback is more and isothermal recovery is less at low $T_{p}$ while springback is less and isothermal recovery is more at high $T_{p}$, the overall shape fixity of SMP first increases and then decreases as the programming temperature is varied. There exists an optimum value of programming temperature ($T_{p}$) at which shape fixity is maximum for a fixed pre-deformation and stress-relaxation time. The optimal $T_{p}$ can guide users to tune $T_{p}$ and $T_{g}$ depending on the application. To obtain maximum shape fixity at any ambient temperature, the $T_{g}$ of SMP should be such that optimum $T_{p}$ equals the ambient temperature. Here, the shape fixity is defined as a multiplicative split of instantaneous and gradual shape fixity. The shape fixity is also dependent on stress-relaxation time. It increases from 55% to 83.5% when relaxed for 0 and 20 min, respectively. The rate of increase in shape fixity with stress relaxation time is very small after 45 min and almost becomes 0 after 100 min. The $T_{p}$ values corresponding to maximum shape fixity are found to be 25.5 and 19 °C for 10% and 40% per-deformation, respectively, and 45 min stress relaxation. A constitutive model capable of predicting large strains and shape fixity based on stress and structural relaxation time is integrated and material parameters are determined. To reduce the experimentation costs and time, numerous thermomechanical cycles are simulated at closed intervals with varying programming temperature, stress relaxation time, and programming strain. The values of optimal temperature and saturation time for various cases of programming strains are evaluated using simulations. The SMP can achieve maximum shape fixity of 92.25% when the programmed at $T_{p}=23$ °C with 100 min stress relaxation time. It is observed that when the stress relaxation time is sufficiently large, the shape fixity is maximized and becomes independent of programming strain.

## Figures and Tables

**Table 1:** Material parameters of the constitutive model for shape memory polymers.

Parameter	Notation	Value
Glass Transition Temperature	$T_{g}$	42.9 °C (4 °C/min)
WLF first constant	$C_{1}$	19.1
WLF second constant	$C_{2}$	58.0 °C
Structural relaxation time (at $T_{g}$)	$\tau_{S}^{o}$	1573.0 s
Coefficient of thermal expansion in rubbery phase	$\alpha_{r}$	8.38 × $10^{-4}$ °C${}^{-1}$
Coefficient of thermal expansion in glassy phase	$\alpha_{g}$	1.32 × $10^{-4}$ °C${}^{-1}$
Equilibrium network shear modulus	$\mu_{r}$	0.9 MPa
Limiting value of locking stretch	$\lambda_{L}$	1.6
Glassy shear modulus	$\mu_{g}$	168.9 MPa
Bulk modulus	*k*	543.1 MPa
Shear viscosity (at $T_{g}$)	$\eta_{o}$	1.48 × $10^{4}$ MPa-s
Initial shear strength	$s_{o}$	20.17 MPa
Activation parameter	$Q/s_{o}$	72.0 K/MPa
Saturation shear strength ratio	$s_{s}/s_{o}$	0.7
Flow softening parameter	*h*	300.0 MPa

## Data Availability

Data sharing is not applicable to this article.

## Appendix A. One-Dimensional Implementation

To solve for one-dimensional uniaxial tension, the tensor equations are converted to algebraic equations by replacing strain tensors with stretch ratios λ. Let us assume that SMP is deformed only in the longitudinal direction ($e_{1}$) and the lateral directions ($e_{2}$ and $e_{3}$) are stress-free. The deformation results from the pure stretching of an element. Therefore, the off-diagonal terms of the deformation gradient tensors are zero. The diagonal terms can be written as stretch ratios $\lambda_{1}$ and $\lambda_{2}$ as follows:

(A1) $$x_{1}=\lambda_{1}X_{1},\;x_{2}=\lambda_{2}X_{2},\;x_{3}=\lambda_{3}X_{3}$$

Since the lateral directions ($e_{2}$ and $e_{3}$) are symmetric for pure uniaxial tension, the strains in lateral directions are equal. The following equation for stretch ratios in the transverse direction applies:

(A2) $$\lambda_{2}=\lambda_{3}$$

Therefore, the deformation gradient tensor defined in Equation (3) can be expressed in matrix form as:

(A3) $$F=\left[\begin{array}{ccc} \lambda_{1} & 0 & 0\\ 0 & \lambda_{2} & 0\\ 0 & 0 & \lambda_{2} \end{array}\right]$$

Similarly, the deformation gradient tensors of other intermediate configurations can be expressed as:

(A4) $$F_{M}=\left[\begin{array}{ccc} \lambda_{M_{1}} & 0 & 0\\ 0 & \lambda_{M_{2}} & 0\\ 0 & 0 & \lambda_{M_{2}} \end{array}\right],F_{e}=\left[\begin{array}{ccc} \lambda_{e_{1}} & 0 & 0\\ 0 & \lambda_{e_{2}} & 0\\ 0 & 0 & \lambda_{e_{2}} \end{array}\right]$$

Using the isochoric property of $F_{p}$ (det$(F_{p})=1$) and the isotropic property of $F_{T}$, the deformation gradient tensors can be expressed as:

(A5) $$F_{p}=\left[\begin{array}{ccc} \lambda_{p} & 0 & 0\\ 0 & \frac{1}{\sqrt{\lambda_{p}}} & 0\\ 0 & 0 & \frac{1}{\sqrt{\lambda_{p}}} \end{array}\right],F_{T}=\left[\begin{array}{ccc} \lambda_{T} & 0 & 0\\ 0 & \lambda_{T} & 0\\ 0 & 0 & \lambda_{T} \end{array}\right]$$

The volumetric deformation, deviator of left Cauchy-green tensor and its principal invariant corresponding to the mechanical component of deformation can be obtained by substituting Equations (A4) and (A5) into Equations (4) and (5):

(A6) $$\begin{array}{cc} \; & J_{M}=\lambda_{M_{1}}\lambda_{M_{2}}^{2}\\ \; & {\bar{b}}_{M}=\left[\begin{array}{ccc} \lambda_{M_{1}}^{\frac{4}{3}}\lambda_{M_{2}}^{-\frac{4}{3}}\; & 0 & 0\\ 0 & \lambda_{M_{1}}^{-\frac{2}{3}}\lambda_{M_{2}}^{\frac{2}{3}} & 0\\ 0 & 0 & \lambda_{M_{1}}^{-\frac{2}{3}}\lambda_{M_{2}}^{\frac{2}{3}} \end{array}\right]\\ \; & {\bar{I}}_{M_{1}}=\lambda_{M_{1}}^{\frac{4}{3}}\lambda_{M_{2}}^{-\frac{4}{3}}+2\lambda_{M_{1}}^{-\frac{2}{3}}\lambda_{M_{2}}^{\frac{2}{3}} \end{array}$$

Similarly, the volumetric deformation, deviator of left Cauchy-green tensor and its principal invariant corresponding to the elastic component of mechanical deformation can be obtained by substituting Equations (A4) and (A5) into Equations (4) and (5):

(A7) $$\begin{array}{cc} \; & {\bar{b}}_{e}=\left[\begin{array}{ccc} \lambda_{e_{1}}^{\frac{4}{3}}\lambda_{e_{2}}^{-\frac{4}{3}}\lambda_{p}^{-2}\; & 0 & 0\\ 0 & \lambda_{e_{1}}^{-\frac{2}{3}}\lambda_{e_{2}}^{\frac{2}{3}}\lambda_{p} & 0\\ 0 & 0 & \lambda_{e_{1}}^{-\frac{2}{3}}\lambda_{e_{2}}^{\frac{2}{3}}\lambda_{p} \end{array}\right]\\ \; & {\bar{I}}_{e_{1}}=\lambda_{e_{1}}^{\frac{4}{3}}\lambda_{e_{2}}^{-\frac{4}{3}}\lambda_{p}^{-2}+2\lambda_{e_{1}}^{-\frac{2}{3}}\lambda_{e_{2}}^{\frac{2}{3}}\lambda_{p} \end{array}$$

The total volumetric deformation *J* can be expressed as:

(A8) $$J=J_{M}J_{T}=\lambda_{M_{1}}\lambda_{M_{2}}^{2}\lambda_{T}^{3}$$

The rate of inverse right Cauchy–Green tensor of plastic deformation is derived by putting Equation (A5) into Equation (5) as follows:

(A9) $$\dot{\bar{{\left(C_{p}\right)}^{-1}}}=\left[\begin{array}{ccc} -\frac{2{\dot{\lambda }}_{p}}{\lambda_{p}^{3}} & 0 & 0\\ 0 & {\dot{\lambda }}_{p} & 0\\ 0 & 0 & {\dot{\lambda }}_{p} \end{array}\right]$$

### Appendix A.1. Thermal Deformation

The thermal component of total deformation is governed by Equation (24). We obtain the following equation if $J_{T}$ is replaced with $det(F_{T})$ in Equation (24):

(A10) $$\lambda_{T}^{3}=1-\alpha_{r}\left(T_{o}-T_{f}\right)-\alpha_{g}\left(T_{f}-T\right)$$

Since the variable $\lambda_{T}$ can be expressed in the form of *T* and $T_{f}$, it can be omitted from the variable set by replacing it with Equation (A10):

### Appendix A.2. Stress Equations

The Cauchy stresses given in Equation (15) are substituted with deformation tensors and principal invariants obtained above.

(A11) $$\begin{array}{cc} \sigma_{vol} & =\frac{1}{J}k\left(J_{M}-1\right)I\\ \; & =\frac{k}{\lambda_{M_{1}}\lambda_{M_{2}}^{2}\lambda_{T}^{3}}\left(\lambda_{M_{1}}\lambda_{M_{2}}^{2}-1\right)\left[\begin{array}{ccc} 1 & 0 & 0\\ 0 & 1 & 0\\ 0 & 0 & 1 \end{array}\right] \end{array}$$

As in Equation (A11), the volumetric component of the stress is the same in all directions.

(A12) $$\begin{array}{cc} \sigma_{dev}^{h} & =\frac{1}{J}\mu_{r}\frac{\lambda_{L}}{\lambda_{chain}}L^{-1}\left(\frac{\lambda_{chain}}{\lambda_{L}}\right)\left({\bar{b}}_{M}-\frac{1}{3}{\bar{I}}_{M_{1}}I\right)\\ \; & =\frac{\mu_{r}}{\lambda_{M_{1}}\lambda_{M_{2}}^{2}\lambda_{T}^{3}}\frac{\lambda_{L}}{\lambda_{chain}}L^{-1}\left(\frac{\lambda_{chain}}{\lambda_{L}}\right)\left[\begin{array}{ccc} \lambda_{M_{1}}^{\frac{4}{3}}\lambda_{M_{2}}^{-\frac{4}{3}}\; & 0 & 0\\ 0 & \lambda_{M_{1}}^{-\frac{2}{3}}\lambda_{M_{2}}^{\frac{2}{3}} & 0\\ 0 & 0 & \lambda_{M_{1}}^{-\frac{2}{3}}\lambda_{M_{2}}^{\frac{2}{3}} \end{array}\right]\\ \; & -\frac{\mu_{r}}{3}\frac{\lambda_{L}}{\lambda_{chain}}L^{-1}\left(\frac{\lambda_{chain}}{\lambda_{L}}\right)\left(\frac{\lambda_{M_{1}}^{\frac{4}{3}}\lambda_{M_{2}}^{-\frac{4}{3}}+2\lambda_{M_{1}}^{-\frac{2}{3}}\lambda_{M_{2}}^{\frac{2}{3}}}{\lambda_{M_{1}}\lambda_{M_{2}}^{2}\lambda_{T}^{3}}\right)\left[\begin{array}{ccc} 1 & 0 & 0\\ 0 & 1 & 0\\ 0 & 0 & 1 \end{array}\right] \end{array}$$

The deviatoric component of stress corresponding to the hyperelastic branch is given in Equation (A12). By manipulation, it can be shown that the trace of $\sigma_{dev}^{h}$ is 0. Furthermore, it can be shown that components of the diagonal matrix follows:

(A13) $$\sigma_{dev_{11}}^{h}=-\frac{1}{2}\sigma_{dev_{22}}^{h}=-\frac{1}{2}\sigma_{dev_{33}}^{h}$$

A similar result is obtained for the matrix $\sigma_{dev}^{ve}$ in Equation (A15).

(A14) $$\begin{array}{cc} \; & \sigma_{dev}^{ve}=\frac{1}{J}\mu_{g}\left({\bar{b}}_{e}-\frac{1}{3}{\bar{I}}_{e_{1}}I\right)\\ \; & =\frac{\mu_{g}}{\lambda_{M_{1}}\lambda_{M_{2}}^{2}\lambda_{T}^{3}}\left[\begin{array}{ccc} \lambda_{e_{1}}^{\frac{4}{3}}\lambda_{e_{2}}^{-\frac{4}{3}}\lambda_{p}^{-2}\; & 0 & 0\\ 0 & \lambda_{e_{1}}^{-\frac{2}{3}}\lambda_{e_{2}}^{\frac{2}{3}}\lambda_{p} & 0\\ 0 & 0 & \lambda_{e_{1}}^{-\frac{2}{3}}\lambda_{e_{2}}^{\frac{2}{3}}\lambda_{p} \end{array}\right]\\ \; & -\frac{\mu_{g}}{3}\left(\frac{\lambda_{e_{1}}^{\frac{4}{3}}\lambda_{e_{2}}^{-\frac{4}{3}}\lambda_{p}^{-2}+2\lambda_{e_{1}}^{-\frac{2}{3}}\lambda_{e_{2}}^{\frac{2}{3}}\lambda_{p}}{\lambda_{M_{1}}\lambda_{M_{2}}^{2}\lambda_{T}^{3}}\right)\left[\begin{array}{ccc} 1 & 0 & 0\\ 0 & 1 & 0\\ 0 & 0 & 1 \end{array}\right] \end{array}$$

The total Cauchy stress tensor is given by:

(A15) $$\sigma =\sigma_{vol}+\sigma_{dev}^{h}+\sigma_{dev}^{ve}$$

### Appendix A.3. Flow Rule

The flow rule of plastic deformation given by Equation (20) is:

(A16) $$\begin{array}{cc} \; & -\frac{1}{2}F_{M}\dot{\bar{\left({\left(C_{p}\right)}^{-1}\right)}}F_{M}^{T}b_{e}^{-1}={\dot{\gamma }}_{p}\frac{\sigma_{dev}^{ve}}{∥\sigma_{dev}^{ve}∥}\\ \; & \end{array}$$

To reduce the number of variables, we replace $\lambda_{e}$ in terms of $\lambda_{M}$ and $\lambda_{p}$ using Equation (2).

(A17) $$\lambda_{M_{1}}=\lambda_{e_{1}}\lambda_{p},\;\;\lambda_{M_{2}}=\frac{\lambda_{e_{2}}}{\sqrt{\lambda_{p}}}$$

The $b_{e}^{-1}$ can be simplified using Equation (A17) as follows:

(A18) $$b_{e}^{-1}=\left[\begin{array}{ccc} \frac{\lambda_{p}^{2}}{\lambda_{M_{1}}^{2}} & 0 & 0\\ 0 & \frac{1}{\lambda_{p}\lambda_{M_{2}}^{2}} & 0\\ 0 & 0 & \frac{1}{\lambda_{p}\lambda_{M_{2}}^{2}} \end{array}\right]$$

First, we simplify the left side of Equation (A16) by substituting Equations (A4), (A9), (A17) and (A18) into it.

(A19) $$-\frac{1}{2}F_{M}\dot{\bar{\left({\left(C_{p}\right)}^{-1}\right)}}F_{M}^{T}b_{e}^{-1}=\left[\begin{array}{ccc} \frac{{\dot{\lambda }}_{p}}{\lambda_{p}} & 0 & 0\\ 0 & -\frac{1}{2}\frac{{\dot{\lambda }}_{p}}{\lambda_{p}} & 0\\ 0 & 0 & -\frac{1}{2}\frac{{\dot{\lambda }}_{p}}{\lambda_{p}} \end{array}\right]$$

To simplify the right-hand side of Equation (A16), the property of the deviatoric stress tensor is used. The trace of the deviatoric stress tensor is zero. Furthermore, the symmetry in directions two and three makes the stress components equal. For simplicity, let a be the first diagonal component of $\sigma_{dev}^{ve}$. It can be expressed as follows:

(A20) $$\sigma_{dev}^{ve}=\left[\begin{array}{ccc} a & 0 & 0\\ 0 & -\frac{a}{2} & 0\\ 0 & 0 & -\frac{a}{2} \end{array}\right]$$

The norm of $\sigma_{dev}^{ve}$ can be evaluated as follows:

(A21) $$\begin{array}{cc} ∥\sigma_{dev}^{ve}∥ & ={\left(\sigma_{dev}^{ve}:\sigma_{dev}^{ve}\right)}^{\frac{1}{2}}\\ \; & =\sqrt{\frac{3}{2}}\left|a\right| \end{array}$$

Thus,

(A22) $$\frac{\sigma_{dev}^{ve}}{∥\sigma_{dev}^{ve}∥}=\left[\begin{array}{ccc} \sqrt{\frac{2}{3}}sgn\left(a\right) & 0\\ 0 & -\frac{1}{2}\sqrt{\frac{2}{3}}sgn\left(a\right) & 0\\ 0 & 0 & -\frac{1}{2}\sqrt{\frac{2}{3}}sgn\left(a\right) \end{array}\right]$$

Combining Equations (A19) and (A22), we obtain the same equation for all three diagonal components, given as:

(A23) $${\dot{\lambda }}_{p}=\sqrt{\frac{2}{3}}{\dot{\gamma }}_{p}\lambda_{p}\;sgn\left(\sigma_{dev_{11}}^{ve}\right)$$

The plastic strain rate ${\dot{\lambda }}_{p}$ should be non-zero during unloading, isothermal recovery, and thermal recovery. Usually, for two-element Maxwell models, ${\dot{\lambda }}_{p}$ is considered zero for the unloading step. Since there are three elements here, the evolution of the plastic strain is not proportional to the sign of the total stress σ but to deviatoric stress in the viscoelastic branch $\sigma_{dev}^{ve}$. When SMP is unloaded, and the external load is removed, σ becomes zero, but the viscoelastic branch is compressed ($\sigma_{dev}^{ve}\leq 0)$ due to a hyperelastic spring in the upper branch, as in Figure 2. This makes $sgn(\sigma_{dev_{11}}^{ve})=-1$. Using the above arguments, the plastic stretch rate is given as:

(A24) $${\dot{\lambda }}_{p}=\left\{\begin{array}{cc} \sqrt{\frac{2}{3}}{\dot{\gamma }}_{p}\lambda_{p}, & \;Loading\\ \sqrt{\frac{2}{3}}{\dot{\gamma }}_{p}\lambda_{p}, & \;Stress\;relaxation\\ -\sqrt{\frac{2}{3}}{\dot{\gamma }}_{p}\lambda_{p}, & \;Unloading\\ -\sqrt{\frac{2}{3}}{\dot{\gamma }}_{p}\lambda_{p}, & \;Isothermal\;recovery\\ -\sqrt{\frac{2}{3}}{\dot{\gamma }}_{p}\lambda_{p}, & \;Heated\;recovery \end{array}\right.$$

## Appendix B. Solution for 1D Equations

The variables appearing in stress equations are $\lambda_{M_{1}}$, $\lambda_{M_{2}}$, $\lambda_{p}$, *s*, *T* and $T_{f}$. In order to obtain the value of these variables for different steps in the thermomechanical cycle, differential equations have to be formed. The differential equations are obtained using evolution equations and some external conditions. This must be performed in such a way that the number of equations equals the number of variables.

The stress tensors have three diagonal components $\sigma_{11}$, $\sigma_{22}$ and $\sigma_{33}$ for the uniaxial case. Because of symmetry in the lateral direction $\sigma_{22}=\sigma_{33}$. We can write $\sigma_{11}$ and $\sigma_{22}$ as a function of the variables $\lambda_{M_{1}}$, $\lambda_{M_{2}}$, $\lambda_{p}$, *s*, *T* and $T_{f}$.

(A25) $$\begin{array}{cc} \; & \sigma_{11}=\sigma_{11}\left(\lambda_{M_{1}},\lambda_{M_{2}},\lambda_{p},s,T,T_{f}\right)\\ \; & \sigma_{22}=\sigma_{22}\left(\lambda_{M_{1}},\lambda_{M_{2}},\lambda_{p},s,T,T_{f}\right) \end{array}$$

Hence, the differential of the stress components can be expressed as:

(A26) $$\begin{array}{cc} {\dot{\sigma }}_{11}= & \frac{\partial \sigma_{11}}{\partial \lambda_{M_{1}}}{\dot{\lambda }}_{M_{1}}+\frac{\partial \sigma_{11}}{\partial \lambda_{M_{2}}}{\dot{\lambda }}_{M_{2}}+\frac{\partial \sigma_{11}}{\partial \lambda_{p}}{\dot{\lambda }}_{p}+\frac{\partial \sigma_{11}}{\partial s}\dot{s}+\frac{\partial \sigma_{11}}{\partial T}\dot{T}+\frac{\partial \sigma_{11}}{\partial T_{f}}\dot{T_{f}}\\ {\dot{\sigma }}_{22}= & \frac{\partial \sigma_{22}}{\partial \lambda_{M_{1}}}{\dot{\lambda }}_{M_{1}}+\frac{\partial \sigma_{22}}{\partial \lambda_{M_{2}}}{\dot{\lambda }}_{M_{2}}+\frac{\partial \sigma_{22}}{\partial \lambda_{p}}{\dot{\lambda }}_{p}+\frac{\partial \sigma_{22}}{\partial s}\dot{s}+\frac{\partial \sigma_{22}}{\partial T}\dot{T}+\frac{\partial \sigma_{22}}{\partial T_{f}}\dot{T_{f}} \end{array}$$

Equations above can be used to solve ${\dot{\lambda }}_{M_{1}}$ and ${\dot{\lambda }}_{M_{2}}$ as follows:

(A27) $${\dot{\lambda }}_{M_{1}}=\frac{b_{1}c_{2}-b_{2}c_{1}}{a_{1}b_{2}-a_{2}b_{1}},\;{\dot{\lambda }}_{M_{2}}=\frac{c_{1}a_{2}-c_{2}a_{1}}{a_{1}b_{2}-a_{2}b_{1}}$$

where

$$\begin{array}{cc} \; & a_{1}=\frac{\partial \sigma_{11}}{\partial \lambda_{M_{1}}},\;b_{1}=\frac{\partial \sigma_{11}}{\partial \lambda_{M_{2}}},\;c_{1}=\frac{\partial \sigma_{11}}{\partial \lambda_{p}}{\dot{\lambda }}_{p}+\frac{\partial \sigma_{11}}{\partial s}\dot{s}+\frac{\partial \sigma_{11}}{\partial T}\dot{T}+\frac{\partial \sigma_{11}}{\partial T_{f}}\dot{T_{f}}-{\dot{\sigma }}_{11}\\ \; & a_{2}=\frac{\partial \sigma_{22}}{\partial \lambda_{M_{1}}},\;b_{2}=\frac{\partial \sigma_{22}}{\partial \lambda_{M_{2}}},\;c_{2}=\frac{\partial \sigma_{22}}{\partial \lambda_{p}}{\dot{\lambda }}_{p}+\frac{\partial \sigma_{22}}{\partial s}\dot{s}+\frac{\partial \sigma_{22}}{\partial T}\dot{T}+\frac{\partial \sigma_{22}}{\partial T_{f}}\dot{T_{f}}-{\dot{\sigma }}_{22} \end{array}$$

The lateral direction is always stress-free during the thermomechanical cycle, therefore the following condition holds for all steps in the thermomechanical cycle:

(A28) $${\dot{\sigma }}_{22}=0$$

It is convenient to write ${\dot{\lambda }}_{M_{2}}$ in the form of ${\dot{\lambda }}_{M_{1}}$ by using Equation (A26) as follows:

(A29) $${\dot{\lambda }}_{M_{2}}=-\frac{\frac{\partial \sigma_{22}}{\partial \lambda_{M_{1}}}{\dot{\lambda }}_{M_{1}}+\frac{\partial \sigma_{22}}{\partial \lambda_{p}}{\dot{\lambda }}_{p}+\frac{\partial \sigma_{22}}{\partial s}\dot{s}+\frac{\partial \sigma_{22}}{\partial T}\dot{T}+\frac{\partial \sigma_{22}}{\partial T_{f}}\dot{T_{f}}}{\frac{\partial \sigma_{22}}{\partial \lambda_{M_{2}}}}$$

In the case of loading, the strain rate ${\dot{\lambda }}_{M_{1}}={\dot{\lambda }}_{o}$ can be calculated directly from the SMP stress–strain curve. Therefore, ${\dot{\sigma }}_{11}$ is not needed to solve the differential equations. Likewise, ${\dot{\lambda }}_{M_{1}}=0$ during the stress-relaxation step since the strain is fixed. For simulation purposes, it is assumed that the stress of $\sigma_{11}$ decays to 0 within 10 ms at the end of the stress relaxation step. Thus, the stress rate ${\dot{\sigma }}_{11}$ for the unloading step is known. ${\dot{\sigma }}_{11}$ is 0 for the isothermal recovery step and the heated recovery step.

(A30) $$\left\{\begin{array}{cc} {\dot{\sigma }}_{11}\neq 0,\;{\dot{\lambda }}_{M_{1}}={\dot{\lambda }}_{o} & Loading\\ {\dot{\sigma }}_{11}\neq 0,\;{\dot{\lambda }}_{M_{1}}=0 & Stress\;relaxation\\ {\dot{\sigma }}_{11}=\frac{\Delta \sigma }{\Delta t},\;{\dot{\lambda }}_{M_{1}}\neq 0 & Unloading\\ {\dot{\sigma }}_{11}=0,\;{\dot{\lambda }}_{M_{1}}\neq 0 & Isothermal\;recovery\\ {\dot{\sigma }}_{11}=0,\;{\dot{\lambda }}_{M_{1}}\neq 0 & Heated\;recovery \end{array}\right.$$

### Appendix B.1. Differential Equations

The set of differential equations for the thermomechanical cycle follows:

1. ${\dot{\lambda }}_{p}=\left\{\begin{array}{cc} \sqrt{\frac{2}{3}}{\dot{\gamma }}_{p}\lambda_{p}, & \;Loading,\;Stress\;relaxation\\ -\sqrt{\frac{2}{3}}{\dot{\gamma }}_{p}\lambda_{p}, & \;Unloading,\;Isothermal\;recovery,\;Heated\;recovery \end{array}\right.$
2. $\dot{s}=h\left(1-\frac{s}{s_{s}}\right){\dot{\gamma }}_{p}$
3. $\dot{T}=\left\{\begin{array}{cc} 0, & Loading,\;Stress\;relaxation,\;Unloading,\;Isothermal\;recovery\\ {\dot{T}}_{exp} & Heated\;recovery \end{array}\right.$
4. $\dot{T_{f}}=\frac{1}{\tau_{S}}\left(T-T_{f}\right)$
5. ${\dot{\lambda }}_{M_{1}}=\left\{\begin{array}{cc} {\dot{\lambda }}_{o}, & Loading\\ 0, & Stress\;relaxation\\ \frac{b_{1}c_{2}-b_{2}c_{1}}{a_{1}b_{2}-a_{2}b_{1}}, & Unloading,\;Isothermal\;recovery,\;Heated\;recovery \end{array}\right.$
6. ${\dot{\lambda }}_{M_{2}}=-\frac{\frac{\partial \sigma_{22}}{\partial \lambda_{M_{1}}}{\dot{\lambda }}_{M_{1}}+\frac{\partial \sigma_{22}}{\partial \lambda_{p}}{\dot{\lambda }}_{p}+\frac{\partial \sigma_{22}}{\partial s}\dot{s}+\frac{\partial \sigma_{22}}{\partial T}\dot{T}+\frac{\partial \sigma_{22}}{\partial T_{f}}\dot{T_{f}}}{\frac{\partial \sigma_{22}}{\partial \lambda_{M_{2}}}}$

### Appendix B.2. Initial Conditions

The SMP is initially undeformed and in equilibrium at t=0. At the starting point of the iterations, the variables are:

(A31) $$\begin{array}{cc} \; & \lambda_{M_{1}}=1,\;\lambda_{M_{2}}=1,\;\lambda_{p}=1,\;\\ \; & s=s_{o},\;T=T_{o},\;T_{f}=T_{o} \end{array}$$

The initial condition for the stress relaxation step is the ending condition for the loading step. Similarly, for other steps, the initial condition is taken from previous steps. This is necessary to solve each step and obtain a continuous variable value throughout the thermomechanical cycle. The differential equations can be solved using an incremental time integration approach or using ODE solvers in MATLAB.
